# Trust matters: a cross-cultural comparison of Northern Ghana and Oaxaca groups

**DOI:** 10.3389/fpsyg.2015.00661

**Published:** 2015-05-20

**Authors:** Cristina Acedo-Carmona, Antoni Gomila

**Affiliations:** Evocog Group, Associated Unit to IFISC (UIB-CSIC), Department of Psychology, University of the Balearic IslandsPalma de Mallorca, Spain

**Keywords:** trust, cooperation, social network, human evolution, ethnic group, cross-cultural analysis

## Abstract

A cross-cultural analysis of trust and cooperation networks in Northern Ghana (NGHA) and Oaxaca (OAX) was carried out by means of ego networks and interviews. These regions were chosen because both are inhabited by several ethnic groups, thus providing a good opportunity to test the cultural group selection hypothesis. Against the predictions of this approach, we found that in both regions cooperation is grounded in personal trust groups, and that social cohesion depends on these emotional bonds. Moreover, in agreement with Fiske's notion of “evolved proclivities,” we also found two distinct kinds of trust networks, one for each region, which vary in terms of the degree of ethnic interrelation. This pattern suggests that social cohesion increases when environmental resources are scarce.

## Introduction

Multiple agent-based models of the evolution of human sociality have tried to pin down the reasons why cooperation is biologically advantageous –kin selection (Hamilton, 1964; Trivers, 1971; Dawkins, 1976), group selection (Boyd and Richerson, 1990; Wilson and Sober, 1994; Bergstrom, 2002), direct or indirect reciprocity (Trivers, 1971; Axelrod, 1984; Caporael et al., 1989). Other models have focused on the human inclination to punish selfish behavior –strong altruism (Boyd et al., 2003, 2010; Gintis et al., 2003, 2008; Bowles and Gintis, 2004). To test some of the previous hypotheses, authors have used methods such as game theory (Henrich et al., 2004, 2006; Hoffman et al., 2007; Marlowe et al., 2008; Ermisch et al., 2009; Gächter and Herrmann, 2009; Dal Bó and Fréchette, 2011). However, even when some of the previous studies use cross-cultural data, they need further anthropological evidence, in order to find out whether their assumptions hold in fact.

Similarly, in this paper we want to contribute to test a particularly influential recent version of group selection theory: the cultural group selection hypothesis (CGS) (Boyd and Richerson, 2009; Richerson et al., 2015). This model explains the emergence of human sociality in terms of a shared culture which guarantees group cohesion and cooperation, so that groups which lack this kind of social cement are overcome, or assimilated by those that do. In other words, the groups with more effective cultures will get greater internal cooperation and will be evolutionarily selected. From CGS follows: (1) that members of a group should cooperate equally with any member of the same cultural group, beyond kin, and (2) it predicts regional cultural homogenization in the long run: if there is competition among co-local cultural groups, the most successful one is deemed to prevail according to CGS, either by exclusion of the other or by absorption/assimilation (by imitation of the most successful one, for instance).

In contrast to this explanation, we contend that cooperation beyond kin is made possible by the creation of small groups united by ties of personal trust (Acedo-Carmona and Gomila, 2014a,b, in press). Although trust has received a lot of attention, personal trust has not been included in evolutionary models of cooperation, basically because evolutionary game-theory takes anonymous playing as a central assumption (Acedo-Carmona and Gomila, 2013). Many studies, though, have developed ways to measure trust (Yamagishi, 1998; Glaeser et al., 2000; Miller and Mitamura, 2003; Naef and Schupp, 2009; Wang and Gordon, 2011), despite the difficulty of this challenge, given the complexity and diversity of the elements involved. In addition, such measurements have been focused mostly on the notion of general trust (Yamagishi, 1998; Putnam, 2000; Uslaner, 2002) –the attitude toward any unknown individual– than on personal trust –which arises from previous positive experiences with known individuals, and involves an affective binding and a mutual expectation of reciprocation (Acedo-Carmona and Gomila, 2012). In previous research using economic games, we have demonstrated that personal trust is the factor that brings cooperation to a maximum (Acedo-Carmona and Gomila, 2013, 2014a). Personal trust consists in a feeling of security, the expectation that positive behaviors will come from the members of one's trust circle. This psychological mechanism unconsciously guarantees a pro-social behavior toward the trusted persons.

In parallel, several researchers have tried to understand the constraints that modulate this basic social structure, so that its concrete manifestation in a particular society may vary depending on the relevant forces involved, as suggested by the notion of “evolved proclivities” (Fiske, 2000): while trust networks can be universal, their particular instantiation in each society may vary according to the adaptive challenges involved. Research by Zhou et al. (2005), Stiller and Dunbar (2007) and Roberts et al. (2009), for instance, show that the number of people that can be included in the closest circles has to be small because of cognitive and time limitations, so that differences can be expected if these limitations are not universal. But other constraints can be imagined, as for instance, scarcity of resources. In dire straits, group support seems to be clearly an advantage -and maybe the only way to manage to survive. There is a long tradition of anthropological studies that relate behavioral ecology and culture (White, 1943; Steward, 1955; Harris, 1966; Rappaport, 1979). However, it remains to be shown that the ecological environment may play a role in the configuration of networks of cooperation and, above all, trust.

To address all the above questions, a cross-cultural approach turns out to be mandatory. We decided to compare two different regions, Northern Ghana (NGHA) and the Oaxaca region (OAX), both inhabited by several ethnic groups. Previous research on Ghana (Adams and Plaut, 2003; Adams, 2005) focused on the way social relations are understood, suggesting that a stringent notion of trust is at play, connected to dependability rather than intimacy. For our purposes, we have been more interested in checking social practices than asking about cultural conceptions, but the results are convergent. The cross-cultural comparison of the NGHA-OAX trust-based networks of cooperation, presented in this work, provides relevant evidence to test the following hypotheses:

- Cooperation is structured around social networks of personal trust -not homogeneously within a cultural group; thus, we expect to find that personal trust fosters cooperation in both settings, so that cooperation is not homogeneously distributed within each cultural group. In fact, intergroup cooperation is possible when intergroup trust is found.
- Cultural differentiation (distinct ethnic and linguistic groups in both cases) contributes to strengthening internal bonds; its persistence calls into question the second prediction of CGS, that cultural homogenization is to be expected.
- The psychology of trust is adapted to the needs of the environment: in the most difficult environments, where individuals need more help, more cohesive networks will emerge, that can cross the group boundaries.

Before getting into the details of the study, we provide next some background of the cultural, social, economic, political and historical contexts that underlie these groups, in order to better understand their respective social lives.

## Regions and groups studied in context

### Regions and groups

#### Oaxaca (México)

**Figure 1:**
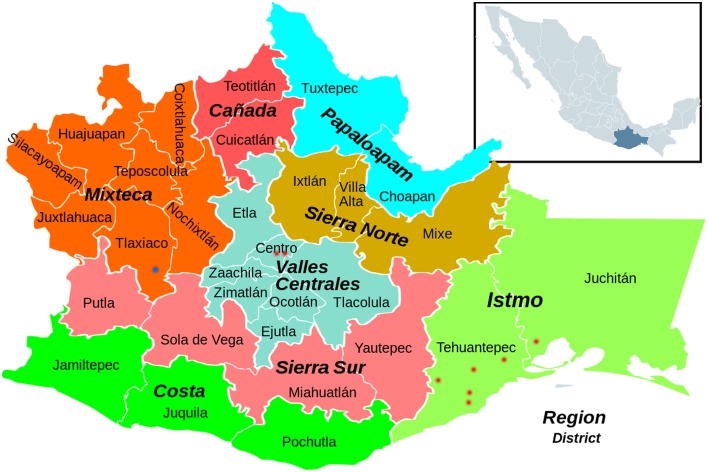
**Situation of Oaxaca, its regions and districts, and the visited locations –red and blue points**. (http://revistatzacualli.blogspot.com.es/. Modified by Cristina Acedo).

Oaxaca is one of the 32 federal entities that form México. It is located in the South of the country, in the southwest of the Tehuantepec Isthmus. It is distributed in 8 regions, which comprise 30 districts (Figure 1). The places and ethnic groups visited for the study were:

- Mixtec region (area Northwest of Oaxaca): *Mixtecs* (Spores, 2008; Joyce, 2010) from Yolotepec de la Paz, Tlaxiaco district, with a population of 151 inhabitants.
- Central Valleys region: *Mestizos* (Chance, 1979) from Oaxaca de Juarez, Center district, with a population of 255,029 inhabitants; and *Zapotecs* (Zeithin, 1990; Joyce, 2010) from Teotitlán del Valle, Tlacolula district, with 4,357 inhabitants.
- Isthmus region: *Chontales* (Oseguera, 2004) of several towns in the District of Tehuantepec, situated both in the highland areas –colonia Marilú, with 411 inhabitants, and San Miguel Ecatepec, with 677 inhabitants, both in Magdalena Tequisistlán, and San Miguel Tenango, with 552 inhabitants–, and in the coastal areas –San Pedro Huamelula, with 2,100 inhabitants and Santiago Astata, with 3,642 inhabitants–; *Zapotecs* (Zeithin, 1990; Campbell, 1993) from Juchitán de Zaragoza, with 74,825 inhabitants, and *Zoques* (Trejo Barrientos, 2006) from San Miguel Chimalapa, with 135 inhabitants, both of them in Juchitán district.

Such diversity of ethnic groups is accompanied by the same diversity of languages or dialects. The participants' languages, in addition to Spanish language, comes from different families of languages: Otomanguean languages (the Zapotec language in different dialects: the main Zapotec in Tequisistlán Valley and the Tehuantepecan Zapotec in Juchitán; and the Mixtec language), Toto-zoquean languages (Mixes languages and the Chimalapas Zoques languages), and the Hokan languages from southern area (Chontal language from Oaxaca, also called Tequistlatecs –highland and coastal dialects).

#### Northern Ghana

**Figure 2:**
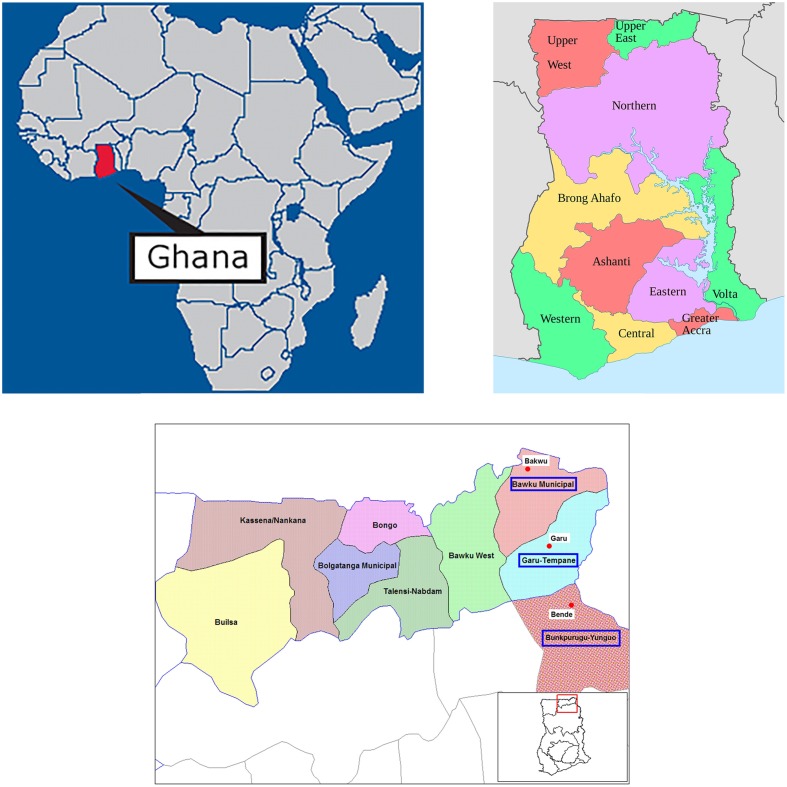
**Situation of Ghana, its districts and the visited areas**. (http://iah241e.wikispaces.com/Ghana) (http://commons.wikimedia.org/wiki/File:Regions_of_Ghana_en.svg) (http://en.wikipedia.org/wiki/File:Upper_East_Ghana_districts.png. Modified by Cristina Acedo).

Ghana has 10 administrative regions, including the regions in the north and the upper-east (Figure 2) where the study was conducted. The places and ethnic groups visited for the study were:

- Upper-East region: fieldwork was carried out in the city of Bawku (district of Bawku, 56,830 inhabitants) and the city of Garu (Garu-Tempane district, 20,802 inhabitants). In the former, we had access to *Kussasis* (Syme, 1932; Hilton, 1962; Awedoba, 1989, 2001), *Frafras* (Hart, 1971), *Bissas* and *Mossis* (Zahan, 1967), and members of groups original from other regions of the country such as the *Ashanti* (Rattray, 1931, 1932), and *Sissalas* and *Waalas* (Wilks, 1989); all of them inhabit the city of Bawku, in the Bawku Municipal district, with 56,830 inhabitants. In Garu, some *Kussasis* also participated.
- Northern region: *Mamprusis* (Drucker-Brown, 1975, 1992; Schlottner, 2000), *Bimobas* (Fussy, 1979; Laari, 1987; Assimeng, 1990), *Konkombas* (Tait, 1961), and *Fulanis* (Oppong, 2002; Tonah, 2005), all of them from Bende, Bunkpurugu-Yunguo district (approximately 5,875 inhabitants).

Participants from Bawku live in an urban environment, and participants from Bende and Garu live in rural areas.

The different languages of all these ethnic groups are part of the set of Nigerien-Congolese languages: the languages Kwa (Ashantis), Gur (Bimobas, Mamprusis, Mossis, Kussasis, Sissalas, Walas, Frafras, Konkombas), Mande (Bissas), and Fulbe (Fulanis). However, each of these branches has resulted in different languages and dialects for each of these groups.

### Historical and cultural contexts

Both areas have a wide diversity of ethnic groups. However, the circumstances in which such multiple groups have emerged and the levels of interaction among groups are different. In both zones, the different cultural groups have coexisted for a long time, but a sense of their own identity has been maintained, despite interacting or sharing a common history. In order to better understand this issue, below, there is a brief review of the historical context in which both social and cultural network structures were created.

#### Oaxaca

The settlements in the current region of Oaxaca followed different stages: agricultural settlements (9000–1500 B.C), “villages” (1500–500 B.C.), urban settlements (500 B.C.–800 A.D.), and “city-states” (750–1521 A.D.). These concentrations were constituted by the union of family lineages (Bandelier, 1982). They grew until the appearance of cities and “cultural civilizations.” Examples of this are the large concentrations of Olmec, Zapotec and Mixtec people in the Middle Pre-classic period (1200–400 B.C.). These City-States became more complex and extended to other areas. They created hierarchical figures who managed different aspects of group life: a maximum governor, advisers, officials (judges and guardians), army heads, province governors, and tax collectors. Therefore, they were societies organized by strict normative regulations.

Although these cities influenced each other, they maintained their own identity, cultural traits, and management. The Mixtec people, for example, were organized into independent Lordships. The same happened with alliances between cities such as Teotihuacan, Monte Alban (Zapotec city), and Tikal (Mayan city) during the Early Classic period (200–600 A.D.), and with the Toltec people in the Late Classic period (600–900 A.D.) and the Early Post-classic period (800/900–1100 A.D.). In the Late Post-classic period (1100–1521/1694 A.D.) a period of wars began, in which some groups invaded others' territory, such as the Mixtec people who forced the Zapotec people toward the Isthmus regions, and the Aztecs –the Mexica people– who were moving toward Oaxaca and Yucatán from the North. However, the Aztecs allowed self-management in the conquered territories in exchange for receiving tributes.

This same autonomy in the management of their own affairs was maintained during the Spanish colonization. Despite the exploitation of indigenous people and the inclusion of new taxes that squeezed their resources, the Spanish Crown in many cases allowed the Indians to keep their self-management, through the communal possession of territory (Reina Aoyama, 2004). The settlers were more interested in the profit from local resources by the intermediation of the noble elite –“caciques”– collecting taxes, than in intervening directly in the management of resources. The colonization pressure positively influenced the revival of ethnic communities because it was a way for the indigenous people to join the community efforts in order to survive and deal with the settlers' pressures.

Throughout the colonial period and the independence of the Republic of Mexico, governors progressively pursued a cultural homogenization, trying to privatize lands and creating municipalities to gain the greatest possible control over resources and people (Nahmad Sitton, 2011). However, far from gaining control over the indigenous people, these policies helped to reaffirm their cultural differentiation and the communal fight to maintain the “*usos y costumbres*” (indigenous customary law) system –their self-management–, that guaranteed participation of the community members in community decisions, including the communal possession of land. In this way, territory, community, and cultural identity merged into the same goal of individuals' subsistence against their exploitation and marginalization by the powerful elite –first, the Spanish nobles, and later, the Creoles and wealthy *Mestizos*. Not in vain, the inherited Mesoamerican beliefs already largely linked group identity to a specific territory (Barabas, 2006, 2008).

In sum, these groups –at first, the pre-Hispanic groups maintaining their cultural specificities in spite of their mutual influence, subsequently, in the Colonial and Republic periods, reaffirming identity as groups of indigenous people, and finally, with communal self-management in municipalities– became the current ethnic groups (Reina Aoyama, 2004). In the case of Oaxaca, these municipalities were also physically separated due to the mountainous nature of these regions and the great climatic diversity. All these elements contributed to the great ethnic diversity that currently appears in the Oaxaca region, where there is a large number of small municipalities with their own cultural specificities regarding clothing, customs, dances, festivals and dialects, despite that also they have common cultural elements due to their long shared history.

#### Ghana

The origin of the ethnic groups in Ghana and their history are not very clear (Southall, 1970; Schildkrout, 1979; Ranger, 1983, 1993; Lentz, 2000). The members of these groups belong to different lineages which at some point coalesced, giving rise to clans. In northern Ghana, in addition to the settlements of acephalous groups of indigenous people, other tribes from other areas also settled.

The North of Africa has had a long history of nomadic tribes moving across the continent and a long tradition of movement of traders. Sub-Saharan migrations due to the trade of gold and salt, and the slave trade caused frequent movements of different groups in the area.

Akan people seem to have migrated from western Africa between the 10th and 12th centuries to the wooded areas of contemporary Ghana and they established small states in the mountain regions. Already in the 10th century, southern Ghana was part of the Asante Empire. These groups were also small “city-states” that, over the centuries, united to form an empire in the 18th century.

In the north of Ghana, it seems that the Mole-Dagbane Kingdoms (Mamprusi, Dagomba and Mossi people) settled between the 13th and 15th centuries, coming from north-east African areas, which dominated the acephalous people installed there. The Kussasi, Frafra, and Sissala people, who also inhabit some areas of northern Ghana, came from west of Sudan and migrated to the area in the 17th century, although other Sissala people seem to be descendants of the Mole-Dagbane groups. The Fulani people came from Niger and Senegal in the 16th century but they migrated to northern Ghana practically at the beginning of the 20th century. The Bissa or Busanga people settled in the White Volta in the 14th century. The Bimoba people are a mixture of the Moba people, who migrated from Burkina Faso in the 17th century, and the Mamprusi and Konkomba people, after being driven to the north by the Mamprusi and Dagomba people. Regarding the Konkomba people, it seems that they are some of the few original groups of these lands. The Waala people, from the city of Wa, capital of the upper-west region, arose from the conjunction in the 17th century of the warrior traditions of the Dagomba and Mamprusi people and Islamic traditions transmitted through small groups of Mande immigrants from Niger. Both the Mamprusi and the Mossi people in the north and the Asante people in the center of Ghana were centralized and hierarchical groups that gained more power, compared to the other mentioned groups, which were acephalous. In these cases, however, the more powerful groups were assimilating the social forms of the groups that were mixed with (Rattray, 1932). There are controversies among authors about these historical data.

In short, all of these groups are the result of the creation of different lineages that were forming their own cultural traits over time. The dynamics of separation and union to form new lineages with their own cultural traits have been continuous. Cultural diversity is observed in their different languages and dialects, beliefs, customs, festivals, etc.; differences either actually based on history or on a common imaginary.

In the 15th century, the Portuguese people exploited the mineral resources of this country and subsequently, the British, French and Dutch people arrived (in the 16th century). In the 17th century, the Asante Confederation unified the groups. Solidarity among groups was appropriate because of the competition to acquire farmlands, control trade routes, and protect each other. In 1901, the areas of northern Ghana became an English Protectorate. The existing rivalries among certain ethnic groups because of fights for territory or slave trade increased with the English colonization, which promoted inequality of power among them and granted certain social and economic courtesies to some ethnic groups in detriment to others. In 1957, the country became independent but the differences before and during the colonial period are the origin of the current inter-ethnic tensions and the cultural reaffirmation of groups.

There are, in this case, groups that also have influenced each other at a cultural level, but the survival pressures and their different allocation of power regarding others have led them to strengthen their ethnic identity.

### Economic context

The resources and economic development of the two countries are also different. Whereas in OAX, the gross domestic product (GDP) per capita by purchasing power parity (PPP) –GDP per capita converted to international dollars using PPP rates–was $16,463 in 2013, in NGHA, it was $3974 (World Bank data).

Whereas OAX is one of the poorest regions of Mexico, it is in a better position than NGHA, because of the productive resources of its environment and its level of economic development.

In OAX, people live mainly from agriculture, as well as tourism, the services sector, crafts, fishing, salt in some coastal areas of the Isthmus region, and recently, wind energy. The climatic diversity in OAX allows cultivation of a wide range of products such as cane sugar, lemon, orange, alfalfa, barley, corn, avocado, pineapple, rice, melon, watermelon, agave, coffee and tobacco. In general, the landscape of OAX offers abundant vegetation and the rains per year are 1550 mm.

Ghana, however, is in the lower middle income countries of the world, with a 24.2% of the population under the threshold of poverty, according to World Bank data for 2012 (http://data.worldbank.org/country/ghana). Its economy has grown, mainly due to improvements in agriculture, the development of the extractive industries, the natural resources of the country (oil, gas, and minerals), the services sector, and manufacturing. However, the north of the country, where we conducted the study, is the poorest region, dominated by the Savannah with large areas of grasses, baobabs, and acacia trees. These arid areas −1.015 mm of rain per year– are used mostly for slash-and-burn farming and livestock. Also, the effects of the Harmattan, a dry wind from the wilderness, which reduces moisture and increases the number of hot days, last about 4 months of the year. Due to the previously mentioned, conflicts because of farmland distribution and the rudimentary and small-scale farming techniques, the inhabitants only have a subsistence economy.

### Social and political structures

Both in OAX and NGHA, relatives play an important social role. However, there are some differences between these countries in the relatives' networks and also in other social structures.

#### Oaxaca

In OAX, the nuclear family –parents and children– is the most widespread form of cohabitation. Sometimes, grandparents or other solitary members of the family can be part of the household, but normally children change their place of residence when they get married. In many cases, the ties between siblings become weaker, especially because of their different place of residence. Other social groups are those arising from the bonds created in the educational, work, and neighborhood contexts in which individuals move, characteristic of societies with a greater number of inhabitants and more complex social organizations.

However, the sense of community is deeply rooted among these groups. Both in urban areas –“*colonias*”– as in the rural area –“municipality”–, people create a sense of community. The community plays a role in the self-management of common affairs (Maldonado Alvarado, 2013). The community is organized through periodic assemblies which all adult members can and must attend–women are increasingly being more accepted in these meetings. The community is also managed through some public service obligations –“t*equios*”– that are required of its members. In the cities, such communities are constituted around the occupied territory, which allows the coexistence of people settled from other municipalities –different cultural traits–, with *Mestizos*.

However, in rural areas, the municipality –administratively established territory– becomes a core of cultural identity, especially as a result of the Spanish colonization and subsequent policies carried out by the Republic of Mexico. The inhabitants of such municipalities are part of different cultural groups, who have maintained certain cultural traits over time and, on other occasions, have developed cultural variations –different dialects, clothing, costumes or dances with certain modifications, etc.– in order to acquire specific hallmarks. The internal community administration regulated by “*usos y costumbres*” has allowed them some kind of self-management. This form of community self-management implies the direct involvement of the whole community, again in periodic assemblies, to decide on common issues. Also, the communal administration of farmlands has given rise to a system of distribution and appropriation of land for its exploitation, while the property remains communal. However, the current gradual inclusion of political parties in municipalities is threatening both the “*usos y costumbres*” system and the communal distribution of land.

All of these forms of self-management and community membership –urban or rural– are accompanied by the implicit normative communal sense that forces its members to comply with their “*tequios*” –community services–, which have a strong tradition. Failure to comply with the mandatory services has negative consequences for the individuals who omit their duties. Therefore, group cohesion has a great normative and authoritative basis. The “*tequio*” has a more extended sense than helping others in order to obtain some future aid if necessary. This sense of required reciprocity is very internalized in individuals, but again, in an unconscious normative sense.

#### Ghana

In NGHA, the extended family still cohabits to a greater extent than in OAX and, in many cases, the family in NGHA includes a greater number of people, as shown in the language, using the same term for different relatives. In addition, in NGHA, the closest relatives follow lineages –matrilineal or patrilineal– and clans, which are cultural ways that gain importance in how society and economy are structured.

In NGHA, monogamy or polygamy are both allowed, depending on own religion. Polygamy occurs very frequently. Relatives that cohabite are generally the extended family, especially in rural areas. The family is composed of the husband and several wives, and the rest of the lineage members –matrilineal or patrilineal– depending on the ethnic group, so that the domestic group may consist of up to 50 people. The most of the ethnic groups in NGHA are patrilineal; only the Akan groups have matrilineal lineages. In urban areas, the relatives' cohabitation tends to be made up of a smaller number of people.

These social structures are also strongly linked to economic aspects, as properties and titles are distributed by lineage, group membership implies group support, and the signs of identity establish the rules for partnerships and marriages. Also the household head –landlord– can decide about the transfer of exploitation rights on the lands the community gives to him, as well as on the management and allocation of tasks among the family members.

The concept of clan usually refers to a group united by ties of kinship or by a common ancestor. In the case of Ghana, the clan can also have a territorial sense and can refer to a lineage, a set of lineages, or groups that occupy a common territory.

Apart from the family and clan, there are other social structures such as the “community” –a group of individuals that resides in a common territory–, that may constitute a clan or several clans; the “heads of clan or lineage” –group of elders– who decide internal clan or lineage issues; the “priests of the Earth” –religious authorities–; and “the chiefs” –who manage the judicial affairs and are mediators in matters with the Government. The “community” assigns the farmlands (Agbosu et al., 2007), and the “heads of clan or lineage” and the “priests of the land” manage communal resources –such as water, hunting, fishing, use of forests, etc.– and oversee rule compliance on such resources (Kotey, 1995).

There are conflicts among some ethnic groups due to the acquisition of administrative power, land rights and historic conflicts among certain ethnic groups that tried to impose their rule on others. The British colonial Government gave greater power to some groups, especially those who already possessed a more hierarchical social structure and power before the colonization, but currently, the groups that were historically acephalous are trying to claim their space of power (Manboah-Rockson, 2004). All of these reasons strengthened ethnic identity.

However, in this case, the groups are linked by a sense of identity and belonging attached to the concepts of lineage and clan, different from the normative cohesion of the OAX groups. Although in both areas, there are some elements in common with respect to the existence of communal ownership of land and ethnic reaffirmation as a way of protection, in NGHA, there is much more interaction between ethnic groups, despite the history of conflicts between some of them. In NGHA, marriages between people coming from different ethnic groups can often be seen. However, in OAX, the different ethnic groups have less contact with each other.

## Methods

### Procedures

Two methods have been used: personal networks of usual cooperators –ego networks–, and interviews. Participants in NGHA were interviewed in English, if possible; if not, they were interviewed in their own language with the help of an interpreter. In OAX, all participants were speakers of Spanish.

#### Ego networks

Ego networks refer to the personal networks of cooperation. In order to obtain them, participants were asked to list the names of people with whom they usually cooperate. They are also asked for the cooperators' ethnic groups, type of relationship between ego and cooperators, type of cooperation they maintain, whether the cooperative relationship is one-sided or mutual (reciprocity), and the trust level they maintain toward cooperators. In Supplementary Materials are presented the forms for the OAX and NGHA groups (Supplementary materials 1, 2).

#### Interviews

Participants were interviewed about trust and cooperation. The interview consisted of 21 questions –Supplementary Material 3 for OAX and 4 for NGHA. The questions focused on the factors involved in the creation, maintenance, and breakdown of trust, seeking the factors that might be universal and those that might be culture-related: questions on what they took into account when deciding to trust someone for the first time; who they trusted more and who were their trustees for secrets; whether it was possible to always trust the family, their meaning of family and their family relationships; the exchanges that required higher levels of trust; what type of situations they considered as a betrayal of trust, whether they would be able to forget such a circumstance, and how they would punish it; their relationships within the same clan; whether they trusted other ethnic groups and their relationships with them; activities carried out in the community to promote trust and integrate new members; importance of reputation in community; the influence of religion or the Government on the attitudes of trust and cooperation toward other individuals; and the level of respondents' feeling of safety, and their values as a group. Among the list of questions, those referring to known trustees concern personal trust, but other questions refer to general trust –such as trusting other ethnic groups in general, or the influence of religion or Government authorities in assuring cooperation, for example.

In order to facilitate the comparison of NGHA and OAX, we chose social practices rather than conceptions. In addition, we tried to adjust as much as possible the equivalence of concepts in translations. Conceptual differences are revealed by the differences in social practices, but we avoid comparing conceptions as such. We also avoided categorizing responses at more abstract clusters. Responses were always mutually exclusive.

### Participants

All participants gave informed consent, following the ethics protocol approved by the Ethics Committee of the University of the Balearic Islands.

#### Ego networks

**Figure 3:**
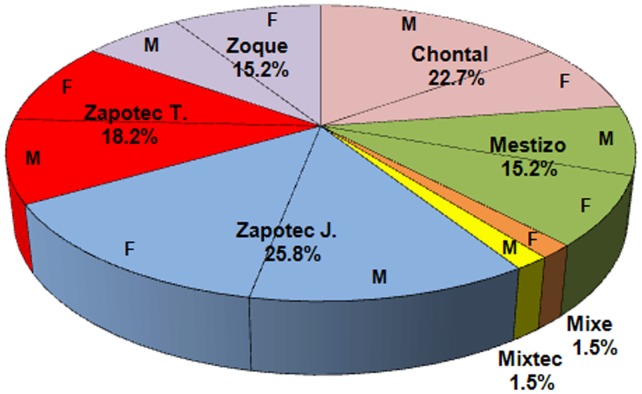
**Distribution of participants by ethnic groups in the method of ego networks of cooperation**. M means male and F means female.

In OAX, 66 persons participated: 34 males (51.5%) and 32 females (48.5%). The participants' ethnic groups are: 15 Chontales (22.7%), 10 Mestizos (15.2%), 1 Mixe (1.5%), 1 Mixtec (1.5%), 17 Zapotecs from Juchitán (25.8%), 12 Zapotecs from Teotitlán del Valle (18.2%) and 10 Zoques (15.2%). The distribution of these ethnic groups by gender is shown in Figure 3.

**Figure 4:**
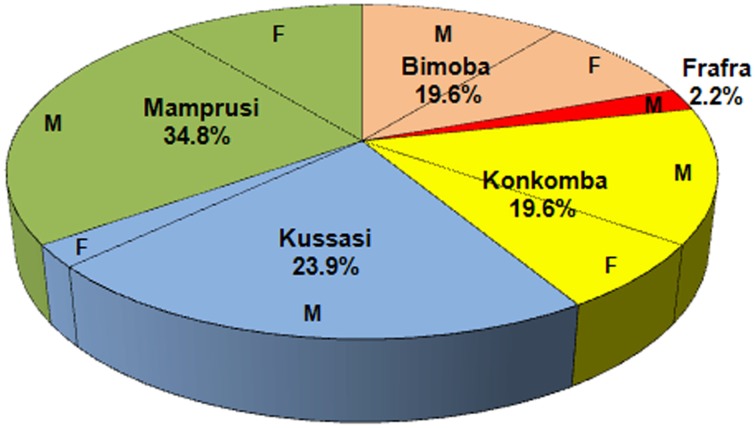
**Distribution of participants by ethnic groups in the method of ego networks of cooperation**. M means male and F means female.

In NGHA, 46 persons participated: 33 males (71.7%) and 13 females (28.3%). The participants' ethnic groups are: 9 Bimobas (19.6%), 1 Frafra (2.2%), 9 Konkombas (19.5%), 11 Kussasis (23.9%), and 16 Mamprusis (34.8%). The distribution of these ethnic groups by gender is shown in Figure 4.

#### Interviews

**Figure 5:**
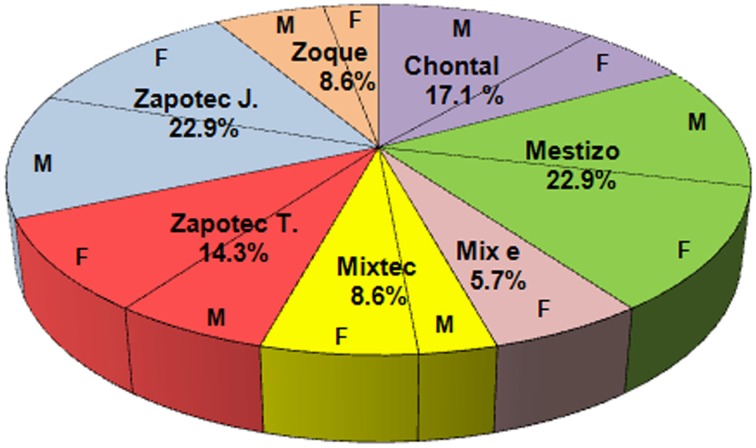
**Distribution of interviewees by ethnic groups and gender**. M means male and F means female.

In OAX, 35 persons were interviewed: 19 males (65.5%) and 10 females (34.5%). The ethnic groups of participants are: 6 Chontales (17.1%), 8 Mestizos (22.9%), 2 Mixes (5.7%), 3 Mixtecs (8.6%), 5 Zapotecs (14.3%), 8 Zapotecs from Juchitán (22.9%), 3 Zoques (8.6%). The distribution of the interviewees by gender is presented in Figure 5.

**Figure 6:**
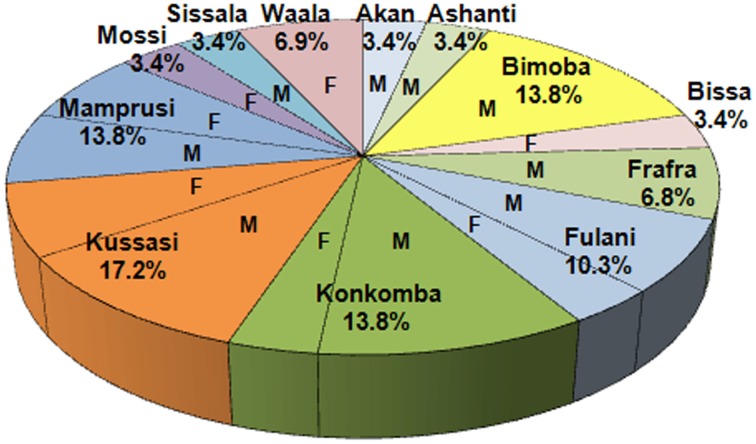
**Distribution of interviewees by ethnic groups**. M means male and F means female.

In NGHA, 29 persons were interviewed: 19 males (65.5%) and 10 females (34.5%). The ethnic groups of participants are: 1, Akan (3.4%), 1 Asante (3.4%), 4 Bimobas (13.8%), 1 Bissa (3.4%), 1 Frafra (3.4%), 3 Fulanis (10.3%), 1 Gruni (3.4%), 4 Konkombas (13.8%), 5 Kussasis (17.2%), and 4 Mamprusis (13.8%), 1 Mossi (3.4%), 1 Sissala (3.4%) and 2 Waalas (6.9%). The distribution of the interviewees by gender is shown in Figure 6.

## Results

We report first the global measures of trust and cooperation for each region: size of personal networks of cooperation, trust levels of usual cooperators, and size of trust circles, and report statistically significant differences between OAX and NGHA for these measures. Given there were some differences in the types of settlements where participants of OAX and NGHA lived (rural, small urban and big urban settlements), the Supplementary Material 5 shows an analysis to rule out that significant differences in trust and cooperation were due to this factor. Once shown statistically that there are significant differences in networks of trust and cooperation between both territories, we consider in more detail the structure and composition of cooperation and trust networks, the levels of general trust, the attitudes toward betrayals of trust and conflicts, and potential influences on trust and cooperation (Supplementary Material 6 shows this information detailed in tables). We distinguish whether the results were obtained through the ego network or the interviews where appropriate.

### Comparison of trust and cooperation between OAX and NGHA

In both territories, the number of usual cooperators reported by the participants in the interviews is not very high. The mean does not exceed 15 people. The mean of usual cooperators is smaller in OAX (9.36) than in NGHA (14.39) (Table 1).

**Table 1:** **Comparison of mean usual cooperators (ego networks)**.

	**Number of usual cooperators**				
	**N**	**Mean**	**Std. Deviation**	**Variance**	**Median**
OAX	66	9.36	5.615	31.527	7.00
NGHA	46	14.39	11.008	121.177	10.50

The participants' trust levels toward their networks of usual cooperators are detailed in **Table 3** by means of the mean of usual cooperators in each trust level. There are more usual cooperators with high trust levels in NGHA than OAX (very high: 5.09 and 3.02; high: 6.59 and 3.27 respectively). There are more usual cooperators with middle trust level in OAX than NGHA (2.38 and 1.65 respectively) (Table 2).

**Table 2:** **Comparison of mean usual cooperators distributed by trust levels (ego networks)**.

**Group**		**Number of cooperators by trust level**				
		**Very.high.trust**	**High.trust**	**Middle trust**	**Low.trust**	**No.trust**
OAX	Mean	3.02	3.27	2.38	0.52	0.18
	N	66	66	66	66	66
	Std. Deviation	2.421	3.698	2.021	1.026	0.763
	Variance	5.861	13.678	4.085	1.054	0.582
	Median	2.50	2.00	2.00	0.00	0.00
NGHA	Mean	5.09	6.59	1.65	0.70	0.30
	N	46	46	46	46	46
	Std. Deviation	4.979	8.145	3.928	1.631	0.840
	Variance	24.792	66.337	15.432	2.661	0.705
	Median	4.00	4.00	0.00	0.00	0.00
Total	Mean	3.87	4.63	2.08	0.59	0.23
	N	112	112	112	112	112
	Std. Deviation	3.812	6.131	2.963	1.305	0.794
	Variance	14.531	37.586	8.777	1.704	0.630
	Median	3.00	3.00	1.00	0.00	0.00
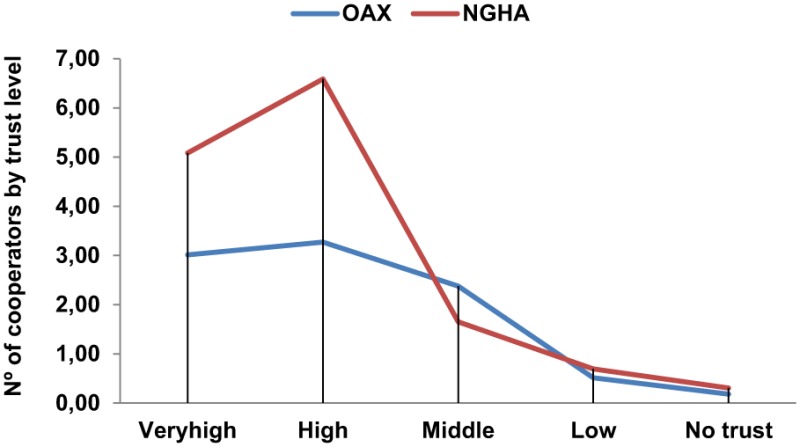						

Although the mean of trustees is higher than the mean of usual cooperators, it doesn't exceed 24 people. Again, the mean of trustees is fewer in OAX (12.50) than in NGHA (23.67) (Table 3).

**Table 3:** **Comparison of mean trustees (interviews)**.

	**Number of trustees**				
	**N**	**Mean**	**Std. Deviation**	**Variance**	**Median**
OAX	34	12.50	19.288	372.015	6.00
NGHA	27	23.67	33.552	1125.769	10.00

There are significant differences –one-tailed Mann-Whitney test– with respect to the number of usual cooperators (*U* = 1135, *z* = −2.27, *N* = 112, *p* < 0.03, *r* = −0.214) and trustees (*U* = 302.5, *z* = −2.28, *N* = 61, *p* < 0.02, *r* = −0.291) between OAX and NGHA: a higher number of people in both cases in NGHA.

In addition, the numbers of usual cooperators that are low or not trusted show no significant differences (*U* = 1514.5, *z* = −0.027, *N* = 112, *p* < 0.5, *r* = −0.002 and *U* = 1404, *z* = −1.256, *N* = 112, *p* < 0.2, *r* = −0.118 respectively) between both regions. But significant differences were found between the number of cooperators with very high (*U* = 1191.5, *z* = −1.95, *N* = 112, *p* < 0.03, *r* = −0.184), high (*U* = 1039.5, *z* = −3.75, *N* = 112, *p* < 0.003, *r* = −0.269) and middle (*U* = 902.5, *z* = −3.75, *N* = 112, *p* < 0.001, *r* = −0.354) trust levels: a higher level of very high and high trusted cooperators in NGHA and middle trusted cooperators in OAX (Table 4).

**Table 4:** **Statistical OAX-NGHA differences in number of cooperators, trustees and trust levels of usual cooperators**.

**Comparison of OAX and NGHA trust (one-tailed Mann Whitney test)**							
**Groups**	**N. coop**.	**N. Trustees**	**Trust level**				
			**Very high trust**	**High trust**	**Middle Trust**	**Low trust**	**No trust**
OAX/NGHA	*U* = 1135 ***p* < 0.03**	*U* = 302.5 ***p* < 0.02**	*U* = 1191.5 ***p* < 0.03**	*U* = 1039.5 ***p* < 0.003**	*U* = 902.5 ***p* < 0.001**	*U* = 1514.5 *p* < 0.5	*U* = 1404 *p* < 0.2

N. coop, number of usual cooperators; N. Trustees, number of trustees; Trust levels respect to usual cooperators, very high, high, middle, low and not trust; p, p-value, significance level.

### Networks of usual cooperators (Ego networks)

The networks of usual cooperators are mostly composed by relatives, friends and neighbors in both territories, but their distribution is different in both regions. In the case of OAX most cooperators are relatives (51.13%), and then friends and neighbors (16.99 and 15.86% respectively). However, in the case of NGHA, most of the usual cooperators are friends (49.24%), followed by relatives (28.33%) and neighbors (11.67%). In both cases, higher trust levels toward usual cooperators also seem to go together with higher levels of reciprocity in their cooperative exchanges.

A bias toward “ethnic endogamy” in cooperation can be found in OAX with respect to NGHA because participants' networks of usual cooperators show fewer individuals of different ethnicity. In OAX just 1 (Mixtec) out of the 7 ethnic groups analyzed had more than the 20% of their usual cooperators of different ethnic group. In NGHA 3 (Kussasi, Mamprusi, Frafra) out of 5 ethnic groups had more than the 20% of their usual cooperators with different ethnicity.

With respect to the types of cooperation most often mentioned with the networks of usual cooperators, there also are differences between OAX and NGHA. The most mentioned in NGHA respect to OAX are advices (30.37 and 17.65%), information (17.76 and 5.37%) and secrets (15.23 and 11.74%). However, OAX refers more to services (18.58 and 7.22%) and lending objects (15.86 and 3.53%) and lending money (12.21 and 7.57%).

There also are differences in the level of reciprocity of the cooperation between the two regions: even when the highest percentage of cooperation among usual cooperators is reciprocal in both territories, this percentage in OAX is higher than in NGHA (84.20 and 74.28%). However, in NGHA there is a higher percentage than in OAX of cases of usual cooperators with no reciprocal cooperation, both when the participant is who receives (13.20 and 7.59%) as the giver (12.53 and 8.20%).

Finally, family plays a greater role in OAX than in NGHA, since for any type of cooperation the percentage of relatives as usual cooperators is higher, except for information exchanges. In NGHA these percentages are divided between family and friends: more relatives than friends for lending money, secrets and services; and more friends than relatives for information, advices, learning and jobs.

### Networks of trustees (interviews)

Circles of trust are mostly composed of relatives in both cases (80.67% in OAX, and 66.22% in NGHA), with more friends in NGHA (7.95 and 13.52%). Relatives' social relevance is also indicated by the proportion of good relationships with relatives (74 and 68.97% respectively) and the proportion of interviewees that declare to trust always the family (51.42 and 44.83% respectively). Relatives are also the preferred recipients of secrets (71.93 and 70.82% respectively), but in NGHA we don't find participants who declared not to tell their secrets to anybody, while in OAX we find the 15.28% of participants.

On the other hand, there is a higher proportion in NGHA than in OAX of participants who declare having good relationships with their nearest network of people: same clan (78.13%) in NGHA; and extended family (62.22%), “*compadres*” (63.64%) and community members (65.75%), in OAX. Besides, in OAX more people declare having little relationship with these networks than in NGHA (28.89 and 12.33% respectively). Together with the higher proportion of interethnic cooperation, these data suggests that in NGHA trust and cooperation extends beyond family. As a matter of fact, family is a more encompassing structure in NGHA. People in NGHA mention more frequently than in OAX the meanings of lineage –“identity and origins” (14.05 for 2.44% in OAX)–, unity of persons –“be together” (26.57 and 3.66% respectively)–, and to a lesser extent, mutual help (29.70 and 24.38%), as relevant dimensions of family. In OAX, on the contrary, family involves an emotional dimension: “something valuable” (12.19 and 9.38% respectively), “give love” (7.32 and 1.56%), and components just mentioned in OAX: “respect” (2.44%), “happiness” (3.66%), “pride” (1.22%). Thus, in NGHA family extends to a greater number of individuals –a lineage– for mutual help.

### General trust (interviews)

In NGHA there is more interaction among ethnic groups than in OAX: the 57.78% of participants in OAX declare not having contact with other ethnic groups but nobody says so in NGHA. Accordingly, more interaction in NGHA translates into a higher proportion of answers of having good (12.35%) and bad (19.35%) relationships with other ethnic groups than in the OAX sample (8.89 and 11.10% respectively). However, the 17.25% of NGHA answers doesn't show clearly how the relationships are with other ethnic groups.

The greater interaction among ethnic groups in NGHA than in OAX has to do with a higher proportion of people who declare trusting other ethnic groups in absolute terms (44.4 and 35.29% respectively). However, the percentage of answers not trusting other ethnic groups is also higher in NGHA respect to OAX (22.22 and 14.71%). The mistrust is also implicit in the expression “it depends” that has a higher proportion in OAX than in NGHA (38.24 and 30.56%).

But a way to clearly measure general trust is the attitude toward unknown people. Although the decision of trusting for the first time in both countries is mostly based on the other's behavior (47.95% in OAX and 48% in NGHA), greater signs of general trust are found again in NGHA. There are a higher percentage of answers in NGHA than OAX of “trust in advance” (18 and 2.74%) and relying on “appearance” (14 and 1.37%). However, in OAX “references” are more needed than in NGHA (15.07 and 12%), where many declare “not be able to trust in advance” (21.92%) nobody says so in NGHA.

As regards newcomers, the NGHA interviewees show an attitude more open than those from OAX. In NGHA, there is a larger number of responses than in OAX that refer to offering a special treatment to the newcomer (20.44% for 9.67%), having meetings and sharing activities (17.18% for 12.89%), communicating (12.90% for 9.68%), giving donations (16.14% for 12.90%), sharing (2.14% for 0), and transmitting the group values and norms (6.46% for 1.07%) with them. In the case of OAX, however, these differences in the percentages with respect to the above-mentioned answers are replaced by attitudes such as “do nothing” (7.53%), the simple coexistence (4.30%) or just know the new person (7.53%).

With respect to the activities to foster trust, in both areas, meetings (45.26% in OAX and 43.65% in NGHA) and shared activities (34.75% in OAX and 29.58% in NGHA) were mentioned. Again, helping has a higher proportion of answers in NGHA than OAX (7.04 and 2.10%), but “doing nothing” has a higher proportion of answers in OAX than NGHA (9.47 and 4.22%). However, a difference can be found with respect to the kinds of meetings that foster trust: festive meetings are mentioned more frequently in OAX than NGHA (26.31 and 15.48%), and religious meetings and rituals more mentioned in NGHA (religious meetings: 8.45% in NGHA and 5.27% in OAX; rituals: 5.64%, just in NGHA).

Additionally, in NGHA there is a higher percentage of affirmative answers on the influence of religion (89.65%) in the level of cooperation than OAX (47.06%) -a factor thought to foster general trust-. Similarly, the Government is attributed a greater role in promoting cooperation in NGHA than in OAX (91.67 and 51.61% respectively).

### Betrayal of trust and conflicts (interviews)

OAX has a lower percentage of people who forget the betrayal of trust (20% for 51.72% in NGHA), and a greater number of people who punish the betrayal of trust (86.35% for 52.73%).

Consistent with the centrality of mutual help, NGHA shows a higher percentage of answers that mentioned the lack of help as a betrayal of trust (19.14% for 3.28% in OAX) or as a reason to break agreements (8.51%). In OAX, on the contrary, gossip is mentioned more frequently as a betrayal of trust (16.40% for 4.25%). Trust betrayal has more consequences in OAX than in NGHA, involving direct punishment (18.17% for 9.10%), and punishment by avoidance (68.18% for 43.63%). Conversely, in NGHA is more frequently answered not punishing the betrayal of trust (25.46% for 4.55%), and proposing measures of approximation (21.81% for 9.10%).

In the same sense, it is also observed more communication (48.10% for 42.97%), less mediation (15.19% for 29.83%), more “forgive and forget” (10.12%) and more attitudes of self-assessment (rectify: 8.86%; understand the problem: 8.86%) as conflict solving strategies in NGHA than in OAX. By contrast in OAX, people mention more passive measures (8.77%) and just “some way” of approach (11.40%).

### Influences on trust and cooperation (interviews)

The different economic situation in both regions, seem to play an indirect role on trust and cooperation, through the different sort of needs of the members of each group. Thus, in OAX only the 10.26% of participants say they feel insecure, while in NGHA, this percentage rises to 43.33%. Similarly, economic fears appear in 12.73% of responses in NGHA, but none in OAX; security fears are mentioned by 17.84% in NGHA, but just 5.12% in OAX; fear about health appears in 12.75% of NGHA respondents, but only in 2.57% from OAX. Security fears in NGHA are related to inter-ethnic conflicts (differences of power, land distribution, etc.), and thefts. In general, fears in NGHA have to do with economic scarcity. This pressing nature of necessity in NGHA also appears in the higher number of responses which explicitly mention the value of mutual help (29.42%, for 5.63% in OAX) as a way to keep trust. This connection between trust and greater necessity is also manifested in that the economic exchanges are thought to require high levels of trust in NGHA (22.85%, for 14.70% in OAX).

Similarly, the different level of available resources of each region is also reflected in the role of reputation. While the importance of reputation is very high in both regions (97.14% in OAX and 93.10% in NGHA), the reasons to gain reputation are different: related to help, in NGHA; related to norm compliance, in OAX. Here reputation comes from “being considered a community member” (34.10% in OAX, 12.50% in NGHA); while in NGHA, good reputation is more frequently associated than in OAX with “a greater level of help received” (27.10% for 9.09%), and with “being respected and trusted” (33.34% for 13.64%), as a way to warrant future support.

## Discussion and conclusion

This study supports the hypothesis that small groups are the basic structure around which networks of trust and cooperation organize. Across different regions and even diverse social groups, a common pattern can be discerned –a small-sized trust circle– as a sort of “evolved proclivity” (Fiske, 2000) to make cooperation possible, as our first hypothesis contends. Despite the ethnic heterogeneity of the regions compared, a similar link between trust and cooperation was found. Against the first prediction of cultural group selection theory, cooperation does not take place homogeneously across cultural groups, but is structured around small groups of trusting people. Against the second prediction of cultural group selection theory, trust reinforces social cohesion, playing the role of an inertial force toward social diversity, which goes against the assimilation process of different groups into the most successful one. Cultural diversity does not disappear in the long run, but it is sustained by the dynamics of cooperation, which does not need to align with culture, as per our second hypothesis.

Our results also support our third hypothesis that the efficacy of the trust network is related to the environmental demands the group faces, among other possible constraints, so that differences are to be expected in the particular arrangement this basic social structure will take in each case. In fact, our results suggest two different types of trust networks corresponding to each region, relative to environmental scarcity and the corresponding degree of group cohesion found:

### Type 1

In OAX, trust circles seem to involve less people, and cooperation is also circumscribed to less people. In particular, high-trust cooperators are almost exclusively relatives, which share affection. General trust is also lower: cooperation takes place among same group members, reluctance to actively incorporate new group members is higher, trust betrayal is not forgotten, and requires punishment. Efforts are not made toward remedying conflicts.

### Type 2

By contrast, in NGHA, both personal trust networks (trustees) and usual cooperators are bigger, with higher proportions of highly trusted usual cooperators. Cooperation also extends to a greater number of people, both from the same lineage and clan, but also from other ethnic groups. General trust is higher: more trustees among other ethnic groups, newcomers are integrated and conflicts solved through communication and active measures of approximation.

These differences can be explained by reference to the different levels of economic and ecological scarcity: subsistence economy and few resources in NGHA, and environmental richness in OAX. Thus, it is observed how in NGHA, where the environmental conditions are harder, there are social networks of trust extended to a higher number of individuals, as a strategy that fosters greater cooperation and hence, guarantees greater chances of survival. Trust networks of type 2 are particularly effective in this sense. In addition to larger trust circles, the NGHA cooperation networks have higher levels of trust. Besides, the circles of cooperation are extended to more different people, thereby ensuring higher chances of survival. At the same time, such persons in NGHA are also more willing to increase trust circles –more active behavior to integrate new people– and to decrease the chances of breaking bonds –greater willingness to forget the betrayal of trust and less willingness to punish it.

In addition, it has also been shown that groups in NGHA have more internalized the assistance to other group members in their system of values –they mentioned more frequently “help” as a necessary requirement to keep trust relationships and as a beneficial effect of having a good reputation. This is consistent with previous research in Ghana, which also found the importance of mutual help in friendship, and the awareness of the risks incurred when trust extends beyond relatives (Adams and Plaut, 2003; Adams, 2005; Acedo-Carmona and Gomila, 2014b).

The type of trust network found in OAX, on the other hand, may have to do with the historical past events. In OAX, ethnic diversity is the outcome of a history in which different groups created their cultural identities over centuries, in spite of being in contact along periods of conquest and/or migratory movements. With the Spanish Colonization and Republic, the groups strengthened their identity and social enclosure as a way to protect their territorial rights and face the marginalization from the established powers. This may have pushed the communities to stay put, in spite of the syncretic transformations of their respective cultures by the Spanish hegemony. Together with this broad common culture, ethnic and linguistic diversity have persisted, against the predictions of cultural group selection theory.

Another factor contributing to keeping with group diversity was may have to do with the orography of the area. Mountainous separation between villages added further difficulty to intergroup exchanges. In addition, cultural differentiation was also kept because of the expression of the “commonality” of municipalities as a group hallmark (Gerrero Osorio, 2013; Nava Morales, 2013) to create association, internal organization and better defend territory (Aquino Zacarías, 2013).

NGHA, on the other side, has a long history of different cultural settlements regardless of national borders, with continuous migration and trading with other groups. However, in this case, groups have interacted more both during British Colonization and after Independence. Conflicts among different cultural groups settled in the same country already existed and with the British Colonization became more intense. This also led to the intensification of ethnic diversity to defend territories and administrative powers, similarly as in OAX.

Taken together, the regions studied are examples of how the unity and association into social groups, is more cohesive when necessary to face contexts of scarcity and lack of power. While the cultural identity of the groups studied was affected by the colonization processes they respectively experienced, their respective patterns of cooperation, grounded in trust networks, provide the cement to keep them diverse –even if more open to intergroup exchange in one case than in the other.

Even when cultural identity of the smaller ethnic groups allowed them to create some way of “emotional” ties that drives them to act together more frequently, trust groups are more basic pillars of cooperation. The psychology of personal trust might be more fostered, however, by emotional figures such as those of lineage and clan of NGHA, more attached to values of belonging, identity and group support than by the regulatory figures of OAX.

A further level of analysis, which we haven't been able to carry out given the insufficient number of participants for this goal, would consist in comparing in more detail the different ethnic groups within each of the studied regions. Still, some relevant features can be discerned. Despite there are more conflicts among ethnic groups in NGHA than in OAX, higher levels of trust among members of different groups are found among them than in OAX, maybe due to the fact that inter-ethnic contact fosters personal trust –in OAX interaction among ethnic groups is rare. Only the Mixtecs from OAX happen to cooperate with non-Mixtecs in a significant proportion, while in NGHA it's more common to cooperate with members of other ethnic groups.

In general, humans seem to become more individualistic in more developed societies, where the economic levels allow acquiring certain security –absence of survival concerns– because they don't have the same need of others for vital support. Culturally, the expression of this is reinforcing family bonds around the nuclear family. Not being so necessary reinforcing the values of unity and trust, new ties arise with lower levels of trust, which are good enough to provide the required benefits in the short term.

In conclusion, this work supports our hypothesis that small groups of trust and cooperation are the basic, universal, form of sociality. This social form is modulated by the particular environment the group is placed and the cultural strategies developed to foster trust and cooperation beyond such a basic social unit. Comparing the NGHA and OAX groups reveals different strategies in this respect, which have to do with the differences in environmental and historical conditions. The more scarce the resources, the higher the degree of social cohesion needed to sustain the society and the greater the push toward keeping ethnic diversity.

### Conflict of interest statement

The authors declare that the research was conducted in the absence of any commercial or financial relationships that could be construed as a potential conflict of interest.

## Abbreviations

OAX: Oaxaca
NGHA: Northern Ghana.
