# Supplementary material for: Trust matters: a cross-cultural comparison of Northern Ghana and Oaxaca groups
Source: Front Psychol. 2015 May 20;6:661. doi: 10.3389/fpsyg.2015.00661 (PMC4440393; doi:10.3389/fpsyg.2015.00661)
Supplement: Supplementary file 1 [file DataSheet1.PDF]

SUPPLEMENTARY MATERIALS

Supplementary material 1.

Ego network of cooperation in OAX.

REDES DE COOPERACIÓN	NOMBRE Y APELLIDO:	______________	GRUPO ÉTNICO	______
Género______________	Población	______________	Edad	______

	NOMBRE Y APELLIDO	GRUPO ÉTNICO	**TIPO DE RELACIÓN**:<br>**F**: familiar (especificar)<br>**A**: amigo<br>**V**: vecino<br>**COMP**: compañero trabajo<br>**C**: conocido<br>**D**: desconocido	**D:** Cooperador me da<br>**R:** Cooperador recibe de mi<br>**DR:** Cooperador me da y recibe de mi	**TIPO DE COOPERACIÓN:**<br>**O**: préstamo objetos<br>**D**: préstamo dinero<br>**S**: compartir secretos<br>**AS**: ayuda con servicios<br>**I**: Conseguir información útil<br>**C**: consejos<br>**E**: enseñar<br>**CI**: contactos e influencias<br>**OP**: oportunidades de trabajo o mejora social<br>**O**: otros (especificar)	**NIVEL DE CONFIANZA**:<br>**MA**: muy alto<br>**A**: alto<br>**M**: medio<br>**B**: bajo<br>**NC**: ninguna confianza
1						
2						
3						
4						
5						
6						
7						
8						
9						
10						
11						
12						
13						
14						
15						
16						
17						
18						
19						
20						
21						
22						
23						
24						
25						
26						
27						
28						
29						
30						
31						
32						
33						
34						
35						

Supplementary material 2.

Ego network of cooperation in NGHA.

	**EGO'S NETWORK**	**NAME & SURNAME:**		**ETHNIC GROUP:**		
	**Gender**	**Place**		**Age**		
	**NAME & SURNAME**	**ETHNIC GROUP**	**RELATIONSHIP TYPE**:<br>**RT**: relative type (put)<br>**F**: friend<br>**N**: neighbor<br>**CW**: co-worker<br>**A**: acquaintance<br>**U**: Unknown	**G:** cooperator GIVES<br>**R:** cooperator RECEIVES<br>**GR:** cooperator GIVES AND RECEIVES	**COOPERATION TYPE**:<br>**O**: Objects loan<br>**M**: Money loan<br>**S**: share secrets<br>**HS**: Help with services<br>**I**: Get useful information<br>**A**: Advices<br>**T**: Teach<br>**CI**: Get contacts and influences<br>**OP**: Get work or social advancement opportunities<br>**OT**: Other answer (Especify)	**TRUST LEVEL**:<br>**VH**: very high<br>**H**: high<br>**IB**: in between<br>**L** : low level<br>**NT**: no trust
1						
2						
3						
4						
5						
6						
7						
8						
9						
10						
11						
12						
13						
14						
15						
16						
17						
18						
19						
20						
21						
22						
23						
24						
25						
26						
27						
28						
29						
30						
31						
32						
33						
34						
35						

Supplementary material 3.

Interview in OAX.

1. ¿Qué tomas en cuenta a la hora de depositar tu confianza en personas que no conoces?
2. ¿Qué necesitas para mantener en el tiempo tu confianza en personas que conoces?
3. ¿Se puede confiar siempre en la familia? ¿Por qué?
4. ¿En qué personas confías más? Número aproximado.
5. ¿Cuándo consideras que una persona en la que confiabas te ha traicionado?
6. ¿Realizáis actividades que fomenten un ambiente de mayor confianza/acercamiento entre vosotros? ¿Cuáles?
7. ¿Qué tipo de intercambios requieren para ti un mayor nivel de confianza?
8. ¿Cómo castigas a las personas que traicionan tu confianza?
9. ¿Es importante en tu comunidad tener buena reputación? ¿En qué afecta tener buena o mala reputación?
10. ¿Qué formas tenéis de fomentar la reconciliación de los individuos que tienen conflictos entre sí?
11. ¿Es posible olvidar la traición de la confianza? ¿Por qué?
12. ¿Cuál es tu visión de los otros grupos étnicos? ¿Se puede confiar en ellos? ¿Por qué? ¿Tienes una buena relación con ellos? ¿En qué se basa esta relación? ¿Ha sido siempre así?
13. ¿Cómo es necesario ser y cómo ha de comportarse un individuo para ser considerado un buen zapoteca/mixteca/etc…?
14. ¿Tus creencias religiosas te hacen relacionarte más y cooperar más con los demás, incluso con extraños? ¿Por qué motivo?
15. Cuando alguien nuevo entra a formar parte del grupo, ya sea en un contexto familiar o de trabajo, o en la comunidad, ¿existen actividades, costumbres o actitudes compartidas para facilitar esta nueva integración? ¿cuáles son?
16. ¿Te sientes protegido en donde vives o trabajas o tienes miedo de algo?
17. ¿Qué piensas del Gobierno, ayudan con sus políticas o su ejemplo a fomentar la cooperación entre los ciudadanos mexicanos?
18. ¿Qué significa la familia para ti? ¿Cómo son las relaciones entre los miembros de tu familia? ¿Por qué?
19. ¿Cómo son tus relaciones con los miembros de tu familia extensa (tíos, primos, sobrinos…), y con los miembros de tu comunidad y con los miembros de diferentes grupos étnicos? ¿Por qué?
20. ¿Con qué personas confías tus secretos o asuntos personales?
21. ¿Has trabajado alguna vez en un mercado? ¿Cuál? ¿Existe confianza y cooperación entre los trabajadores del mercado? ¿En qué crees que se basa esta confianza o desconfianza?

Supplementary material 4.

Interview in NGHA.

1. What do you have in mind when you decide to trust people that you don't know?
2. What do you need to maintain your trust in people you know?
3. It is possible to always trust the family?
4. Which people do you trust more? Number.
5. When do you consider that one of your trustees has betrayed you?
6. Do you perform activities to foster an atmosphere of greater trust and rapprochement among you?
7. What kind of exchanges requires a higher level of trust?
8. How you punish people who betray your trust?
9. Is it important here to have good reputation? What implications does a good or a bad reputation have?
10. What forms do you have to encourage reconciliation of individuals with conflicts?
11. Is it possible to forget the betrayal (of trust)?
12. What do you think about other ethnic groups? Can you trust them? Do you have a good relationship with them? What is this relationship is based on? Has this relationship always been this way?
13. How should an individual behave to be considered a good Kussasi, Mamprusi, etc...?
14. Do your religious beliefs make you interact and cooperate more with others, even with strangers?
15. When somebody becomes part of a new family or begins to work in a new market are there activities, customs or shared attitudes to facilitate this new integration?
16. Do you feel protected where you live or work, or you are afraid of something?
17. What do you think about Government authorities, do they help with their policies or their example to facilitate the lives of citizens and promote coexistence among all?
18. What does family mean to you? How are the relationships among family members?
19. How are relationships among members within the same clan, among members of different clans, and among members of different tribes?
20. With which people do you trust your secrets or personal issues?
21. Is there trust and cooperation among workers in the market? What do you think this trust or distrust is based on?

Supplementary material 5.

Analysis of trustees and usual cooperators by type of location of participants

Given the heterogeneity of participants respect to location in both countries, it is questioned if the difference in the number of cooperators (Tab. 6), trustees (Tab. 7) and trust levels towards usual cooperators (Tab. 8) distributed by locations can be influenced by this factor. The composition of participants in NGHA includes people dispersed in rural areas (30.67%), others who reside in urban areas of less than 6,000 inhabitants (46.67%) and those living in urban areas of over 50,000 inhabitants (22.67%). In the case of OAX, participants are divided between those who reside in the two types of urban areas mentioned previously (55.45% and 44.55% respectively) (Table 5).

Composition of participants by types of location						
	Ego's networks (N)		Interviews (N)		%	
	OAX	NGHA	OAX	NGHA	OAX	NGHA
Rural areas		16		7	0,00%	30,67%
Urban areas no more than 6,000 inh.	39	25	17	10	55,45%	46,67%
Urban areas more than 50,000 inh.	27	5	18	12	44,55%	22,67%
Total	66	46	35	29	100,00%	100,00%

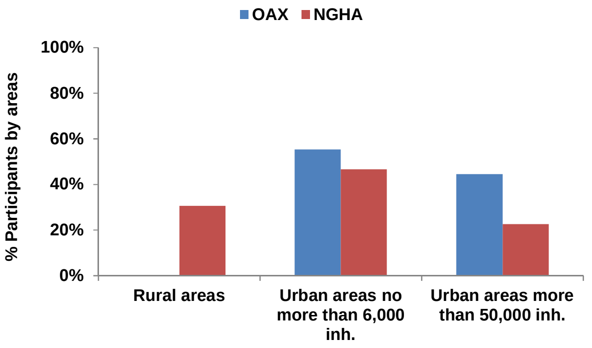

Table 5. Number and proportion of participants by location in NGHA and OAX.

N. cooperators						
	Rural areas		Urban areas			
			No more 6,000 inh.		More than 50,000 inh.	
	OAX	NGHA	OAX	NGHA	OAX	NGHA
Mean		15,06	10,33	10,36	7,96	**32,00**
N		16	39	25	27	5
Std. Deviation		8,054	6,084	6,383	4,612	**19,672**
Variance		64,863	37,018	40,740	21,268	387,000
Median		14,00	8	10,00	7,00	44,00

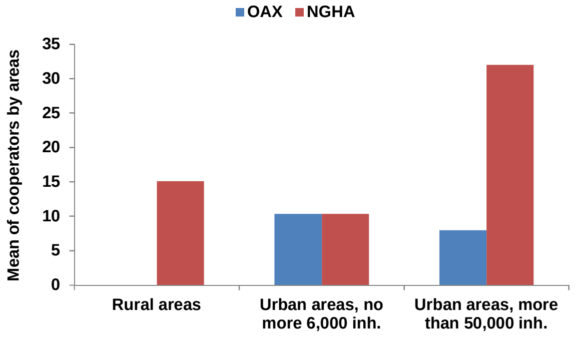

Table 6. Mean of usual cooperators by location in NGHA and OAX (ego networks).

N. trustees						
	Rural areas		Urban areas			
			No more 6,000 inh.		More than 50,000 inh.	
	OAX	NGHA	OAX	NGHA	OAX	NGHA
**Mean**		48,29	12,71	21,88	12,29	10,50
**N**		7	17	8	17	12
**Deviation**		**51,661**	13,990	**29,878**	**23,905**	7,740
**Variance**		2668,905	195,721	892,696	571,471	59,909
**Median**		50,00	9,00	11,00	4,00	10,00

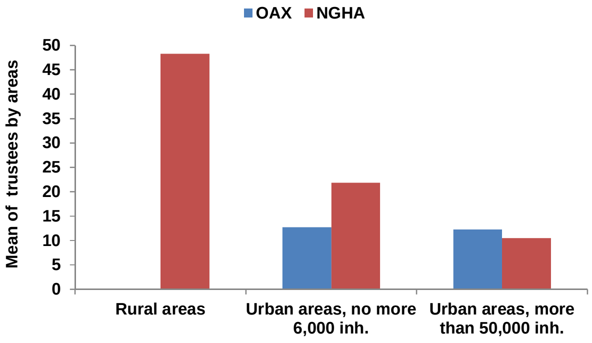

Table 7. Mean of trustees by location in NGHA and OAX (interviews).

Mean of n. cooperators by trust level											
GROUP		Very high trust		High trust		Middle trust		Low trust		No trust	
		OAX	NGHA	OAX	NGHA	OAX	NGHA	OAX	NGHA	OAX	NGHA
**Rural areas**	Mean		4,31		6,38		2,19		1,50		,69
	N		16		16		16		16		16
	Std. Deviation		6,107		3,384		2,428		2,366		1,250
	Variance		37,296		11,450		5,896		5,600		1,563
	Median		2,00		5,50		1,00		,50		,00
**Urban areas no more than 6,000 inhab.**	Mean	3,08	6,00	4,10	4,00	2,62	,32	,36	,04	,18	,00
	N	39	25	39	25	39	25	39	25	39	25
	Std. Deviation	2,860	4,463	4,235	4,601	2,098	,690	,959	,200	,823	,000
	Variance	8,178	19,917	17,937	21,167	4,401	,477	,920	,040	,677	,000
	Median	2,00	5,00	3,00	2,00	2,00	,00	,00	,00	,00	,00
**Urban areas more than 50,000 inhab.**	Mean	2,93	3,00	2,07	20,20	2,04	6,60	,74	1,40	,19	,60
	N	27	5	27	5	27	5	27	5	27	5
	Std. Deviation	1,639	2,550	2,336	17,398	1,891	10,286	1,095	1,673	,681	,894
	Variance	2,687	6,500	5,456	302,700	3,575	105,800	1,199	2,800	,464	,800
	Median	3,00	3,00	2,00	14,00	2,00	1,00	,00	1,00	,00	,00
**Total**	Mean	3,02	5,09	3,27	6,59	2,38	1,65	,52	,70	,18	,30
	N	66	46	66	46	66	46	66	46	66	46
	Std. Deviation	2,421	4,979	3,698	8,145	2,021	3,928	1,026	1,631	,763	,840
	Variance	5,861	24,792	13,678	66,337	4,085	15,432	1,054	2,661	,582	,705
	Median	2,50	4,00	2,00	4,00	2,00	,00	,00	,00	,00	,00

Table 8. Mean of usual cooperators by location and trust levels in NGHA and OAX (ego networks).

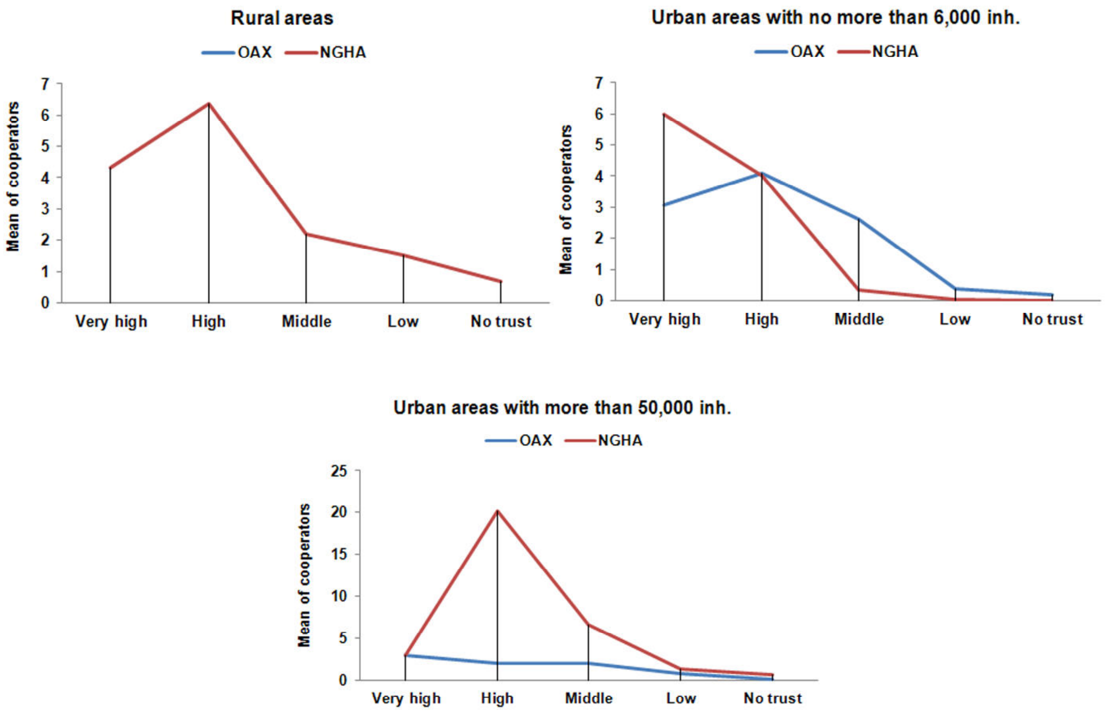

Figure 7. Representation of the participants' mean of usual cooperators distributed by location and trust levels (ego networks).

In OAX, the participants' different settlements doesn't show significant differences –one-tailed Mann-Whitney test– between small and big urban areas in number of trustees, usual cooperators, or cooperators with very high, middle and no trust level. But there are significant differences among these areas in number of usual cooperators with high trust levels ($U = 382$, $z = -1.908$, $N = 66$, $p < 0.03$, $r = -0.234$): more cooperators in small urban areas, and low trust levels ($U = 407.5$, $z = -1.983$, $N = 66$, $p < 0.03$, $r = -0.244$): more cooperators in big urban areas.

In NGHA, we don't find significant differences in the number of trustees according to the area, but there are significant differences in number of usual cooperators among rural and small urban areas ($U = 124$, $z = -2.037$, $N = 41$, $p < 0.03$, $r = -0.318$): more cooperators in rural areas, and among small and big urban areas ($U = 24$, $z = -2.152$, $N = 30$, $p < 0.02$, $r = -0.392$): more cooperators in big urban areas. Respect to the level of trust of usual cooperators, there are significant differences in each of the trust levels between rural and small urban areas (Very high: $U = 131.5$, $z = -1.843$, $N = 41$, $p < 0.04$, $r = -0.287$; High: $U = 101$, $z = -2.660$, $N = 41$, $p < 0.004$, $r = -0.415$; Middle: $U = 83$, $z = -3.512$, $N = 41$, $p < 0.001$, $r = -0.548$; Low: $U = 105.5$, $z = -3.490$, $N = 41$, $p < 0.001$, $r = -0.545$; No trust: $U = 137.5$, $z = -2.939$, $N = 41$, $p < 0.007$, $r = -0.458$): more very high trusted usual cooperators in small urban areas and more high, middle, low and no trusted usual cooperators in rural areas. There are also significant differences in number of high trusted usual cooperators among rural and big

urban areas ($U = 19$, $z = -1.745$, $N = 21$, $p < 0.05$, $r = -0.380$): more in big urban areas, and in the number of usual cooperators in each of the trust levels except the very high one among small and big urban areas (High: $U = 19.5$, $z = -2.410$, $N = 30$, $p < 0.008$, $r = -0.440$; Middle: $U = 34$, $z = -2.040$, $N = 30$, $p < 0.04$, $r = -0.372$; Low: $U = 26.5$, $z = -3.391$, $N = 30$, $p < 0.006$, $r = -0.619$; No trust: $U = 37.5$, $z = -3.216$, $N = 30$, $p < 0.03$, $r = -0.587$): more in big urban areas.

Comparing NGHA and OAX by locations, there are not significant differences in number of trustees and usual cooperators among small urban areas. Among big urban areas, by contrast, even when there are not either significant differences in number of trustees, there are significant differences in number of usual cooperators ($U = 21.5$, $z = -2.412$, $N = 32$, $p < 0.008$, $r = -0.426$): more usual cooperators in NGHA. Among small urban areas, there are not significant differences in number of high, low and no trusted usual cooperators, but there are differences in number of very high trusted usual cooperators ($U = 290$, $z = -2.735$, $N = 64$, $p < 0.004$, $r = -0.341$): more in NGHA, and middle trusted ones ($U = 156.5$, $z = -4.809$, $N = 64$, $p < 0.001$, $r = -0.601$): more in OAX. Among big urban areas there are significant differences in number of high trusted usual cooperators ($U = 17$, $z = -2.663$, $N = 32$, $p < 0.004$, $r = -0.470$): more in NGHA.

Therefore, with respect to trust and cooperation, in OAX there is an effect of the type of participants' locations: a higher level of trust on usual cooperators in small urban areas than in the big ones. In NGHA, this location effect is confusing: there are more usual cooperators in rural and big urban areas than small urban areas; significant differences in trust levels of usual cooperators between rural and small urban areas, for every trust levels: more very high trusted usual cooperators in small urban areas, and more usual cooperators of any other trust levels in rural areas; and significant differences between big and small urban areas in every trust levels except the very high one: more usual cooperators in big urban areas.

However, comparing OAX and NGHA, at big cities, there are more usual cooperators and high trusted usual cooperators in NGHA than OAX. At the small urban areas, there are more very high trusted usual cooperators in NGHA and more middle and low trusted usual cooperators in OAX (Tab. 9).

	Level of trust	Type of location	OAX	NGHA	Significant differences OAX-NGHA
N. trustees (mean)		Rural		48.29	
		Small urban	12.71	21.88	
		Big urban	12.29	10.50	
N. cooperators (mean)		Rural		15.06	
		Small urban	10.33	10.36	
		**Big urban**	**7.96**	**32.00**	**U = 21.5, p < 0.008**
N. usual cooperators by trust levels (mean)	Very high	Rural		4.31	
		**Small urban**	**3.08**	**6.00**	**U = 290, p < 0.004**
		Big urban	2.93	3.00	
	High	Rural		6.38	
		Small urban	4.10	4.00	
		**Big urban**	**2.07**	**20.20**	**U = 17, p < 0.001**
	Middle	Rural		2.19	
		**Small urban**	**2.62**	**0.32**	**U = 156.5, p < 0.001**
		Big urban	2.04	6.60	
	Low	Rural		1.50	
		Small urban	0.36	0.04	
		Big urban	0.74	1.40	
	No trust	Rural		0.69	
		Small urban	0.18	0.00	
		Big urban	0.18	0.30	

Table 9. Significant differences OAX-GHANA by type of locations (ego networks and interviews).

In short, since the number of trustees doesn't change significantly because of participants' location and the number of cooperators changes only in NGHA, the trust levels of usual cooperators differ between the two territories, being higher in NGHA. So, there are significant differences in trust and cooperation regardless of the effect caused by the type of location where participants are settled. If the rural areas in NGHA are added, then more significant differences between the two regions appear. In the next tables (Tab. 10, 11, 12) appears the details of signification of the previously mentioned differences by locations in number of trustees, cooperators and trust levels of usual cooperators respectively.

Comparison OAX & NGHA of n. trustees by area type									
Type of area	OAX			NGHA				OAX-NGHA	
	Mean	Small urban	Big urban	Mean	Rural	Small urban	Big urban	Small urban	Big urban
Rural				48.29		U = 24.5 p < 0.4	U = 24.5 p < 0.08		
Small urban	12.71		U = 97.5 p < 0.06	21.88			U = 32 p < 0.2	U = 42.5 p < 0.08	
Big urban	12.29			10.50					U = 71.5 p < 0.1

Table 10. Statistical differences in number of trustees by locations (interviews).

Type of area	OAX			NGHA				OAX-NGHA	
	Mean	Small urban	Big urban	Mean	Rural	Small urban	Big urban	Small urban	Big urban
**Rural**				15.06		**U = 124 p < 0.03**	U = 21.5 p < 0.07		
**Small urban**	10.33		U = 407 p < 0.06	10.36			**U = 24 p < 0.02**	U = 466 p < 0.4	
**Big urban**	7.96			32.00					**U = 21.5 p < 0.008**

Comparison OAX & NGHA of n. usual cooperators by area types

Table 11. Statistical differences in number of usual cooperators by locations (ego networks).

Trust levels	Type of areas	OAX			NGHA				OAX-NGHA	
		Mean	Small urban	Big urban	Mean	Rural	Small urban	Big urban	Small urban	Big urban
Very high trust	Rural				4.31		**U = 131.5 p < 0.04**	U = 38.5 p < 0.5		
	Small urban	3.08		U = 487 p < 0.4	6.00			U = 38 p < 0.1	**U = 290 p < 0.004**	
	Big urban	2.93			3.00					U = 66 p < 0.5
High trust	Rural				6.38		**U = 101 p < 0.004**	**U = 19 p < 0.05**		
	Small urban	4.10		**U = 382 p < 0.03**	4.00			**U = 19.5 p <0.008**	U = 465.5 p < 0.4	
	Big urban	2.07			20.20					**U = 17 p < 0.004**
Middle trust	Rural				2.19		**U = 83 p < 0.001**	U = 37.5 p < 0.5		
	Small urban	2.62		U = 432.5 p < 0.2	0.32			**U = 34 p < 0.04**	**U = 156.5 p < 0.001**	
	Big urban	2.04			6.60					U = 67.5 p < 0.6
Low trust	Rural				1.50		**U = 105.5 p < 0.001**	U = 36.5 p < 0.4		
	Small urban	0.36		**U = 407.5 p < 0.03**	0.40			**U = 26.5 p < 0.006**	U = 417.5 p < 0.06	
	Big urban	0.74			1.40					U = 51 p < 0.3
No trust	Rural				0.69		**U = 137.5 p < 0.007**	U = 38 p < 0.6		
	Small urban	0.18		U = 526 p < 0.7	0.00			**U = 37.5 p < 0.03**	U = 450 p < 0.3	
	Big urban	0.19			0.30					U = 47 p < 0.2

Comparison OAX & NGHA of number of usual cooperators by trust levels and area types

Table 12. Statistic differences in trust levels of usual cooperators by locations (ego networks).

Supplementary material 6.

Information detailed in tables

	% Cooperators	
	OAX	NGHA
Relative	**51,13%**	28,33%
Friend	16,99%	**49,24%**
Co-worker	6,15%	6,36%
Neighbor	**15,86%**	11,67%
Acquaintance	**8,58%**	3,18%
Unknown	0,49%	0,76%
Chief	0,32%	,0%
Girldfriend	0,16%	,0%
Godmother	0,16%	,0%
Godson	0,16%	,0%
Guardian	,0%	0,45%

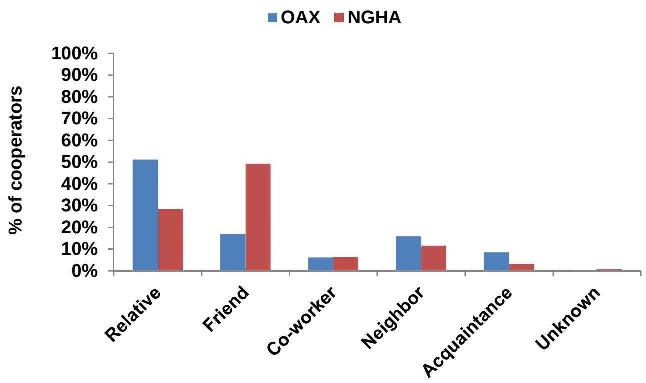

Table 13. Type of relationship of participants with their usual cooperators (ego networks).

% In-group cooperation by ethnic groups						
OAX						
Chontal	Mestizo	Mixe	Mixtec	Zapotec Juchitán	Zapotec Teotitlán	Zoque
97,9%	90,9%	80,0%	52,4%	90,9%	100,0%	95,6%

% In-group cooperation by ethnic groups				
NGHA				
Bimoba	Kussasi	Mamprusi	Frafra	Konkomba
83,3%	51,4%	67,1%	47,7%	95,8%

Table 14. Proportion of in-group cooperation by ethnic groups in OAX and NGHA (ego networks).

	% OAX-NGHA of type of cooperation		% OAX-NGHA comparison of reciprocity by type of cooperation					
			Reciprocity		P.receives		P.gives	
	OAX	NGHA	OAX	NGHA	OAX	NGHA	OAX	NGHA
**Job**	3,73%	3,53%	**2,87%**	2,16%	0,49%	**0,86%**	0,37%	**0,52%**
**Lend objects**	**15,86%**	3,53%	**11,42%**	2,15%	**2,32%**	0,86%	**2,12%**	0,51%
**Lend money**	**12,21%**	7,57%	**11,24%**	6,09%	0,32%	**0,61%**	0,65%	**0,87%**
**Secrets**	11,74%	**15,23%**	10,72%	**12,88%**	0,17%	**0,78%**	0,85%	**1,57%**
**Services**	**18,58%**	7,22%	**15,00%**	4,78%	1,09%	**1,22%**	**2,49%**	1,22%
**Information**	5,37%	**17,76%**	4,59%	**14,37%**	0,47%	**1,74%**	0,31%	**1,65%**
**Advices**	17,65%	**30,37%**	15,49%	**23,05%**	1,37%	**3,83%**	0,80%	**3,49%**
**Learn**	12,06%	**12,62%**	**10,72%**	7,75%	1,01%	**2,70%**	0,34%	**2,17%**
**Contacts**	**2,80%**	2,17%	**2,16%**	1,04%	0,35%	**0,61%**	0,29%	**0,52%**
**Total**	**100,00%**	**100,00%**	**84,20%**	74,28%	7,59%	**13,20%**	8,20%	**12,53%**

Table 15. OAX-NGHA comparison on composition of type of cooperation and reciprocity with usual cooperators. P means participants (ego networks).

	% Reciprocity by levels of trust				
	Very high	High	In between	Low level	No trust
**OAX**	77.3%	69.8%	66.3%	58.8%	64.3%
**NGHA**	76.9%	74.9%	69.7%	53.8%	55.0%

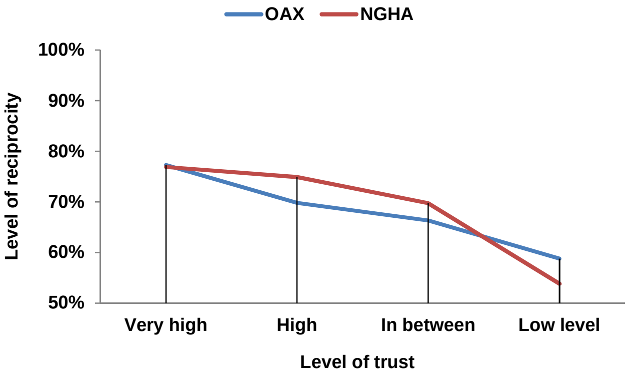

Table 16. Detail and representation of the proportion of reciprocity in cooperative exchanges, according to the trust levels toward usual cooperators (ego networks).

	% OAX-NGHA comparison of type of cooperation by type of relationship																	
Type of relationship	Job		Lend money		Lend objects		Secrets		Services		Information		Advices		Learn		Contacts	
	OAX	NGHA	OAX	NGHA	OAX	NGHA	OAX	NGHA	OAX	NGHA	OAX	NGHA	OAX	NGHA	OAX	NGHA	OAX	NGHA
**Relative**	**1,71%**	**0,73%**	**8,17%**	**4,18%**	**9,56%**	**0,73%**	**7,62%**	**7,40%**	**10,49%**	**3,91%**	**3,42%**	**6,78%**	**10,11%**	**10,69%**	**7,08%**	**4,09%**	**1,40%**	0,69%
**Girldfriend**			0,08%															
**Godmother**			0,08%						0,08%									
**Godson**	0,08%		0,08%		0,08%				0,08%									
**Friend**	0,23%	**1,81%**	1,09%	**2,96%**	**1,48%**	**1,81%**	2,18%	**6,70%**	**2,10%**	**1,39%**	0,54%	**8,44%**	3,89%	**14,37%**	2,10%	**6,10%**	0,39%	0,87%
**Co-worker**	0,39%	0,26%	0,78%	0,26%	**0,47%**	0,26%	0,39%	0,26%	0,78%	0,52%	0,08%	1,23%	0,86%	1,58%	0,31%	0,96%	0,16%	0,09%
**Neighbor**	0,93%	0,65%	1,40%	0,17%	**3,65%**	0,65%	1,40%	0,70%	**3,89%**	0,96%	1,09%	0,87%	2,33%	2,70%	1,79%	0,87%	0,62%	0,26%
**Acquaintance**	0,31%	0,09%	0,47%		**0,62%**	0,09%	0,16%	0,08%	1,17%	0,26%	0,16%	0,44%	0,39%	0,76%	0,70%	0,43%	0,16%	0,26%
**Chief**			0,08%										0,08%		0,08%			
**Unknown**	0,08%									0,17%	0,08%			0,09%		0,18%	0,08%	
**Guardian**								0,09%						0,18%				
**Total**	3,73%	**3,53%**	**12,21%**	7,57%	**15,86%**	3,53%	11,74%	**15,23%**	**18,58%**	7,22%	5,37%	**17,76%**	17,65%	**30,37%**	**12,06%**	12,62%	**2,80%**	2,17%

Table 17. OAX-NGHA comparison of type of cooperation by type of relationship with usual cooperators (ego networks).

% The most trusting people		
	OAX	NGHA
Relatives	**80,67%**	**66,22%**
Friends	**7,95%**	**13,52%**
Co-workers	3,41%	1,35%
God	1,14%	
Godparents	4,55%	
Godchildren	1,14%	
Neighbords	1,14%	
People with whom live		4,06%
People with same language		1,35%
Same group or clan		1,35%
People who believe in you		1,35%
People who help you		1,35%
Men more than women		1,35%
Chief		2,70%
Assembly/Committee		1,35%
Children in the community		1,35%
In travel, mamprusis		1,35%
Women		1,35%

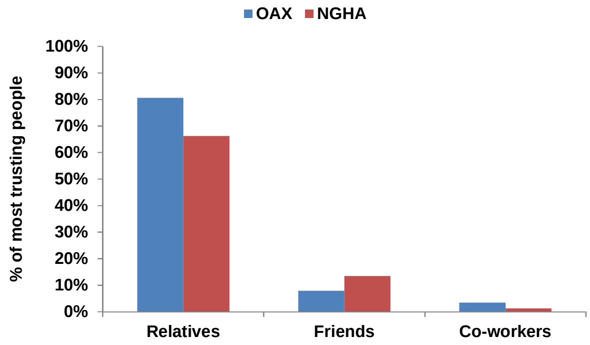

Table 18. Type of relationships of participants with their trustees (interviews).

% Family relationships		
	OAX	NGHA
Good	**74,00%**	**68,97%**
Conflicts	26,00%	31,03%

% Trust always the family		
	OAX	NGHA
Yes	**51,42%**	**44,83%**
No	34,28%	44,83%
Close family, no extended	2,86%	6,89%
It depends	8,58%	3,45%
Own family, no husband's family	2,86%	

Table 19. Details of participants' relationships with their relatives and proportion of participants who trusts always the family (interviews).

% People for secrets		
	OAX	NGHA
**Relatives**	**71,93%**	**70,82%**
**Friends**	7,02%	**15,28%**
**Nobody**	**7,02%**	
**God**	3,51%	1,39%
**Religious people**		1,39%
**Boss**	1,75%	
**Co-worker**	3,51%	
**People of the community**		1,39%
**Chief**		1,39%
**Assembly/Committee**		1,39%
**District director**		1,39%
**Women**		1,39%
**Elders**		1,39%
**Kusasis**		1,39%
**Mossis**		1,39%
**It depends on the secret**	1,75%	
**There are not secrets**	3,51%	

Table 20. People for secrets in percentages (interviews).

% Relationships				
	OAX			NGHA
	Extended-family	"Compadres"	Community	Same clan
**Good**	**62,22%**	**63,64%**	**65,75%**	**78,13%**
**Conflicts**	8,89%	27,27%	21,92%	21,87%
**Little relationship**	**28,89%**		**12,33%**	
**Neutral**		9,09%		

Table 21. Comparison of the participants' relationships with their closest networks of people apart from their close relatives (interviews).

% Meaning of family		
	OAX	NGHA
**Being together**	3,66%	**26,57%**
**Identity and origins**	2,44%	**14,05%**
**Help**	**24,38%**	**29,70%**
**Something valuable**	**12,19%**	9,38%
**Trustful people**	6,09%	4,69%
**Comfortable people**	6,10%	3,12%
**They listen to you**	3,66%	1,56%
**Give love**	7,32%	1,56%
**Children,be alive and happy**	8,54%	4,69%
**Not to be alone**	8,54%	1,56%
**Big influence on the person**	3,66%	1,56%
**The best unit of society**		1,56%
**Friends and neighbors as family**	2,44%	
**Obligations**	3,66%	
**Respect**	2,44%	
**Happiness**	3,66%	
**Pride**	1,22%	

Table 22. Meanings of family for the interviewees.

% Relationships			
		OAX	NGHA
Other ethnic groups	**Good**	**8,89%**	**12,35%**
	**Conflicts**	**11,10%**	**19,35%**
	**Conflicts with some of them**		**26,41%**
	**Neutral**	22,23%	22,89%
	**No contact**	**57,78%**	
	**It depends of heads relationship**		1,75%
	**It is not known**		17,25%

Table 23. Relationships of the interviewees with other ethnic groups.

% Trust			
		OAX	NGHA
Other ethnic groups	**Yes**	**35,29%**	**44,44%**
	**No**	14,71%	22,22%
	**It depends**	38,24%	30,56%
	**They don't know**	8,82%	
	**He doesn't mixes but trust the rest**	2,94%	
	**No trust people with power, traditional people**		2,78%

Table 24. Proportion of interviewees' answers in relation to trust other ethnic groups.

	Decide trust firstly	
	OAX	NGHA
**Behavior**	**47,95%**	**48,00%**
**Trust firstly**	2,74%	**18,00%**
**Appearance**	1,37%	**14,00%**
**References**	**15,07%**	12,00%
**Social Capital**	1,37%	**6,00%**
**Trust reciprocity**	2,74%	
**No possible**	**21,92%**	
**Other's opinion**	1,37%	
**Make questions**	**5,47%**	
**Visit home**		2,00%

Table 25. Comparison of reasons to trust for the first time (interviews).

	Ways of integration	
	OAX	NGHA
Treatment	9,67%	**20,44%**
Meetings & shared activ.	12,89%	**17,18%**
Communication	9,68%	**12,90%**
Give	12,90%	**16,14%**
Behavior	13,99%	8,60%
Introductions	11,84%	7,53%
Learn rules & values	1,07%	**6,46%**
Help	7,53%	7,53%
Nothing	**7,53%**	
Coexistence	**4,30%**	
Know the new person	**7,53%**	
Decide to accept	1,07%	
Trust		1,08%
Share		**2,14%**

Table 26. Ways to integrate new people to the own group, according to the interviewees.

	Activities to foster trust	
	OAX	NGHA
Meetings	**45,26%**	**43,65%**
Shared activities	**34,75%**	**29,58%**
Some behaviors	1,05%	8,46%
Help	2,10%	**7,04%**
Opportunities program	2,11%	
Nothing	9,47%	4,22%
To learn	1,05%	
Go out	1,05%	
Greetings	2,11%	
Introductions	1,05%	
Don't know		2,82%
Know each other		1,41%
Physical contact		1,41%
Share material things		1,41%

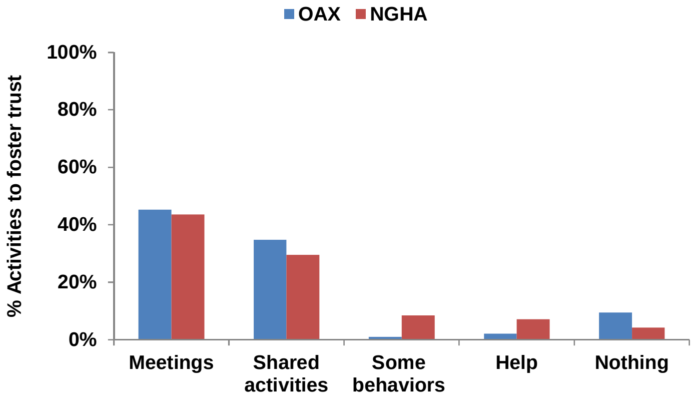

Table 27. Detail of the activities to foster trust, according to the interviewees.

% Types of meetings		
	OAX	NGHA
Festive	**26,31%**	**15,48%**
Religious	**5,27%**	**8,45%**
Visits	2,11%	1,41%
Meetings	10,52%	12,67%
Sportive events	1,05%	
Ritual		**5,64%**

Table 28. Classification of the type of meetings to foster trust mentioned by the interviewees.

% Influences on cooperation (Participants' perception)		
**Religion**	**OAX**	**NGHA**
Yes	**47,06%**	**89,65%**
No	47,06%	10,35%
Sometimes	2,94%	
It depends	2,94%	
**Government**	**OAX**	**NGHA**
Yes	**51,61%**	**91,67%**
No	25,81%	8,33%
Don't know	6,45%	
It depends	16,13%	

Table 29. Comparison of interviewees' perception of religion and Government influence in people's cooperation.

Comparison of trust						
	OAX			NGHA		
**Number of cooperators (mean)**	9.36			14.39		
**Number of trustees (mean)**	12.50			23.67		
**Level of trust (mean of people)**	**Very high**	**High**	**In beween**	**Very high**	**High**	**In beween**
	3.02	3.27	2.38	5.09	6.59	1.65
**Forget betrayal of trust (% people)**	**20.00%**			51.72%		
**Punishment of betrayal of trust (% people)**	**86.35%**			52.73%		

Table 30. OAX-NGHA comparative summary on the mean of usual cooperators, trustees and usual cooperators distributed by trust levels. Also they are enclosed the percentages of respondents who are able to forget and punish the betrayal of trust (ego networks and interviews).

% Ways of betrayal		
	**OAX**	**NGHA**
**Lie**	8,20%	6,38%
**Gossip**	**16,40%**	4,25%
**Not to keep secrets**	**18,02%**	**21,28%**
**Not to like the behavior**	6,55%	8,51%
**Not to help**	3,28%	**19,14%**
**Break agreements**		**8,51%**
**Total percentage**	52,45%	68,07%

Table 31. Comparison of the most frequently reasons of trust betrayal mentioned by interviewees.

% Punishment of trust betrayal		
	OAX	NGHA
**Ways of punishment**	**18,17%**	**9,10%**
Send to authorities	6,82%	7,28%
If they are abusing, gun and kill them	2,27%	
Not to help	2,27%	
Criticize	2,27%	
Not to give work	2,27%	
Not to give nothing	2,27%	
Pay back		1,82%
**Ways of avoidance**	**68,18%**	**43,63%**
Avoid	15,91%	18,18%
Not to trust	25,00%	7,27%
Not to talk	15,91%	12,72%
No greetings		1,82%
Not to invite	2,27%	
Ignore	6,82%	
Don't keep relationship		1,82%
Not to invite home	2,27%	
No secrets		1,82%
**No punishment**	**4,55%**	**25,46%**
**Approach measures**	**9,10%**	**21,81%**

Table 32. Ways of punish trust betrayal adopted by the interviewees.

% Ways of solve conflicts		
	OAX	NGHA
**Communication**	**42,97%**	**48,10%**
**Mediations**	**29,83%**	**15,19%**
**Passive measures**	8,77%	
**Forgive and forget**		10,12%
**Blessing**		1,27%
**Good manners**	4,39%	7,60%
**Approach measures**	11,40%	
**Rectify**		8,86%
**Understand the problem**		8,86%
**Search for the guilty**	0,88%	
**Violence**	1,76%	

Table 33. Ways to solve conflicts used by the interviewees.

% Feeling of security		
**Security**	**OAX**	**NGHA**
Yes	**89,74%**	**53,33%**
No	10,26%	**43,33%**
It depends		3,34%

% Fears		
	OAX	NGHA
**Economic problems**	0,00%	**12,73%**
**Security problems**	5,12%	**17,84%**
**Health problems**	2,57%	**12,75%**
**The unknown**	2,57%	0,00%
**Total**	10,26%	43,32%

% Fears			
OAX		NGHA	
**Economic problems**		**Economic problems**	**29,40%**
		Problems with animals	5,88%
		Problems with house	5,88%
		Problems with farm	5,88%
		Poverty	5,88%
		Not having food	5,88%
**Security problems**	**50,00%**	**Security problems**	**41,18%**
To thieves	50,00%	Thieves at night	5,88%
		Conflicts	11,77%
		Not get to sleep	5,88%
		Bimobas and chieftancy	5,88%
		Be harmed by an unknown	11,77%
**Health problems**	**25,00%**	**Health problems**	**29,42%**
Children become sick	25,00%	Accidents	5,88%
		To die and live	5,88%
		Pains when sun is hot	5,89%
		Mosquitos, snakes, scorpions	11,77%
**The unknown**	**25,00%**	**The unknown**	
The unknown	25,00%		

Tables 34. Feeling of security and detail of the interviewees' fears.

	Keep trust	
	OAX	NGHA
**Behavior**	**42,26%**	**41,18%**
**Communication**	**25,34%**	17,64%
**Help**	5,63%	**29,42%**
**Meeting and shared activities**	5,64%	
**Feel good with trustee**	1,41%	
**Trust reciprocity**	**11,27%**	
**Nothing**	2,82%	
**Coexistence**	1,41%	
**Interaction**	4,22%	
**Not to steal**		1,96%
**Keep promises**		3,92%
**Faith**		3,92%
**Unity**		1,96%

Table 35. Reasons given by interviewees to keep trust along time.

% Trustful exchanges		
	OAX	NGHA
Secrets	13,23%	14,58%
Econ. exchanges	14,70%	**22,85%**
Help	23,54%	20,85%
Feelings & concerns	**17,65%**	8,34%
Respect & coexistence		6,24%
Meetings	5,88%	6,27%
Introduce people		2,09%
Donations	4,41%	4,18%
Personal or valuable objects	7,36%	
Time	1,47%	
Everything	8,82%	
Family	2,94%	
Family activities		2,09%
Marriage		4,15%
Travel exchanges		2,09%
Education		2,09%
Way of interactions		2,09%
Faith		2,09%

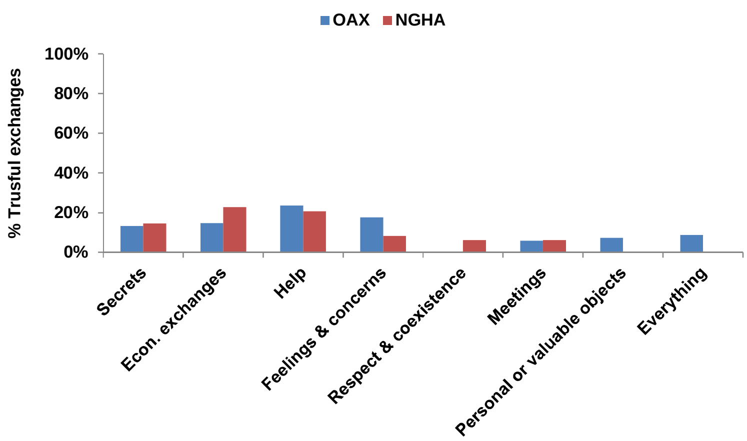

Table 36. Detail of the interviewees' answers on their most trustful exchanges.

% Importance of reputation		
	OAX	NGHA
Yes	**97,14%**	**93,10%**
For others, not for him	2,86%	
No response		6,90%

% Consequences of reputation		
	Good reputation	
	OAX	NGHA
Help	9,09%	**27,10%**
Opportunities	18,18%	18,74%
Inside community	**34,10%**	12,50%
Position (respect & trust)	13,64%	**33,34%**
Relationships with others	6,81%	4,16%
Feelings	4,54%	2,08%
Attributions	4,55%	2,08%
No matter	6,82%	
Benefits to the community	2,27%	

Table 37. Importance of reputation and consequences of having good reputation, according to the interviewees.
